# Genotypic diversity and molecular characterization of DENV-2 in a Peruvian endemic region from 2016 to 2022: displacement of American/Asian genotype

**DOI:** 10.3389/fmicb.2025.1558761

**Published:** 2025-04-28

**Authors:** Yordi Tarazona-Castro, Miguel Angel Aguilar-Luis, Wilmer Silva-Caso, Hugh Watson, Victor Zavaleta-Gavidia, Ronald Aquino-Ortega, Luis J. Del Valle, Jorge Bazan-Mayra, Egma Mayta Huatuco, Juana del Valle-Mendoza

**Affiliations:** ^1^Biomedicine Laboratory, Research Center of the Faculty of Health Sciences, Universidad Peruana de Ciencias Aplicadas, Lima, Peru; ^2^Unidad de Postgrado de la Facultad de Ciencias Biológicas, Universidad Nacional Mayor de San Marcos, Lima, Peru; ^3^Antiviral Research Unit, Evotec ID, Lyon, France; ^4^Dirección Regional de Salud de Cajamarca, DIRESA-Cajamarca, Cajamarca, Peru; ^5^Facultd de Medicina Humana, Universidad Nacional de Cajamarca, Cajamarca, Perú; ^6^Centre d'Enginyeria Biotecnologica i Molecular (CEBIM), Departamento de Ingeniería Química, ETSEIB, Universidad Politěcnica de Catalunya (UPC), Barcelona Tech, Barcelona, Spain; ^7^Barcelona Research Centre in Multiscale Science and Engineering, Universitat Politècnica de Catalunya, BarcelonaTech (UPC), Barcelona, Spain

**Keywords:** arboviruses, acute febrile illness, dengue, phylogenetic, DENV genotype

## Abstract

**Background:**

Dengue is the most prevalent acute febrile disease with serious clinical consequences in the tropical and subtropical regions of Asia and America. In Peru, it represents a significant public health issue due to its hyperendemic nature, with serotype 2 (DENV-2) being the predominant serotype that leads to the most severe clinical manifestations of the disease. This study focuses on the molecular characterization and analysis of the intraserotypic diversity of DENV-2 circulating in the endemic region of Cajamarca.

**Methods:**

A total of 3,967 blood serum samples from patients with acute febrile illness (AFI) were analyzed between 2016 and 2022 to detect DENV and DENV-2 using real-time RT-PCR. The viral envelope (E) gene was then sequenced using the Sanger method. Finally, phylogenetic reconstruction was conducted using the maximum likelihood method.

**Results:**

A total of 32 complete sequences of the envelope gene were obtained, and the phylogenetic and characterization analyses of the amino acid sequences revealed that, during the period from 2016 to 2022, two DENV-2 genotypes circulated: the Am/As genotype and the cosmopolitan genotype in lineages 2 and C, respectively.

**Conclusion:**

Similarly, our findings showed that every studied outbreak was characterized by novel autochthonous variants of the Am/As genotype and by an imported variant of the cosmopolitan genotype; this demonstrates a temporal distribution of intraserotypic variability that indicates the displacement of the Am/As genotype around 2021 and the establishment of the cosmopolitan genotype. The need for ongoing genetic or genomic surveillance of the cosmopolitan virus arises in order to understand its distribution and diversification patterns in Peru.

## 1 Introduction

The disease caused by the dengue virus (DENV) manifests clinically in a range of asymptomatic infections to severe conditions that may lead to death (WHO, 2023a). It is estimated that ~390 million DENV cases each year go unreported (Bhatt et al., 2013). Despite its global distribution, Asia accounts for 70% of cases, while the Americas had the second highest incidence until 2022 (Brady et al., 2012). However, nearly 80% of the global DENV burden was recorded in the Americas in 2023 (WHO, 2023b) due to epidemics, with Brazil contributing to nearly 75% of the American burden (PHAO, 2023). At the South American level, Peru ranked second with 273,684 reported cases and recorded the highest death rate in the Americas (MINSA, 2023; PHAO, 2024).

Peru is an endemic DENV country that has seen an increase from 15,287 cases in 2019 to 63,168 cases by the end of 2022, multiplying more than 4fold by 2023 (MINSA, 2023). This situation has transformed what was, until recently, a seasonal public health problem into a constant health emergency. Climate, population, socioeconomic, vectorial, and viral factors contribute to the ongoing development of DENV outbreaks and endemics in urban, peri-urban, and rural areas (Dostal et al., 2022; Guerra-Gomes et al., 2017).

DENV comprises four serotypes: DENV-1, DENV-2, DENV-3, and DENV-4, with an amino acid variability of 25 to 40% (Guzman and Harris, 2015). Furthermore, its genome consists of a positive-sense, single-stranded RNA molecule of ~10.7 kB that encodes the structural proteins (C capsid, pr/M pre-membrane/membrane, and E envelope) and non-structural proteins (NS1, NS2A, NS2B, NS3, NS4A, NS4B, and NS5; Iglesias and Gamarnik, 2011), as well as a single open reading frame (ORF). The extensive variability of DENV is due to the high rate at which it accumulates mutations, contributing to DENV's genetic and antigenic diversity across all its serotypes and genotypes (Domingo and Holland, 1997).

Each DENV serotype consists of several genotypes, which in turn give rise to lineages and their own variants. Regarding DENV-2, there are five genotypes (Asian 1, Asian 2, cosmopolitan, American, and American/Asian) that are transmitted to humans (Twiddy et al., 2002). Four dengue serotypes circulate in Peru, with DENV-2 being the most frequent. There are two described genotypes from the Americas (American and American/Asian) that were imported between 1995 and 2000 (Montoya et al., 2023; Mamani et al., 2011) until 2019, when the Asian cosmopolitan genotype was introduced for the first time (García et al., 2022).

Some studies report that DENV-1 poses a higher risk of causing severe dengue. In comparison, DENV-2 cases present a lower risk of severe disease relative to DENV-1 and DENV-3. Additionally, it has been reported that viral RNA levels in DENV-1 can be nearly twice as high as those in DENV-2 and DENV-3 cases. In contrast, viral RNA levels in DENV-2 cases are significantly lower compared to DENV-1 and DENV-3 serotypes. Other reports indicate that during primary infection, DENV-3, and during secondary infection, DENV-2, DENV-3, and DENV-4 exhibit a higher risk of severe dengue infections. This highlights the importance of serotype identification for making clinical predictions regarding the severity of infection (Yung et al., 2015; Soo et al., 2016).

The Am/As genotype has predominated in the Americas for decades, contributed to the genetic diversity of dengue, and is associated with outbreaks of severe disease (Amorim et al., 2023). In Peru, this genotype has been the primary cause of endemic transmission since its introduction in the 2000s, with reported circulation from the Amazon to the northern coast. However, in recent years, the emergence and expansion of the cosmopolitan genotype, particularly lineage 5, have been documented in several Latin American regions, including Brazil, Ecuador, and Peru (Amorim et al., 2023; Mir et al., 2014). This phenomenon is significant for public health, as the cosmopolitan genotype has been associated with an increase in the incidence of severe dengue in other regions of the world (Anh et al., 2024).

The DENV molecular epidemiological study in Peru has primarily focused on the hyperendemic regions of Loreto, Piura, and Madre de Dios. To date, no studies have involved the endemic region of Cajamarca. Therefore, this study aims to describe the genetic diversity of outbreaks associated with DENV-2 in the endemic region of northern Peru by conducting phylogenetic analysis and molecular characterization of the E gene of DENV-2 genotypes that circulated from 2016 to 2022.

## 2 Materials and methods

### 2.1 Data availability

The datasets presented in this study can be found in online repositories. The names of the repository/repositories and their accession number(s) can be found in the article/Supplementary material.

### 2.2 Ethical statement

This study was approved by the Institutional Research Ethics Committee of the Regional Health Directorate of Cajamarca in Peru (Official letter No. 4724-2023-GR-CAJ/DRS-ORE-CIEI). Samples were collected as part of the epidemiological surveillance program for acute febrile syndrome in the Cajamarca region, thus exempting it from informed consent. The management and analysis of clinical samples were conducted anonymously, in line with the International Ethical Guidelines for Health-related Research Involving Humans (CIOMS, 2016).

### 2.3 Place of study and patients

A cross-sectional study of DENV outbreaks in Cajamarca, a region in northern Peru, was conducted from 2016 to 2022. Data were actively collected at primary care health centers of DIRESA-Cajamarca during the sampling years, and data analysis was performed retrospectively.

The inclusion criteria comprise all individuals with acute febrile illness (AFI) who visited primary care health centers with a temperature exceeding 38°C for < 7 days, without an identifiable source of infection, and who exhibited at least one of the following signs and symptoms: headache, myalgia, arthralgia, retro-ocular pain, lower back pain, rash, nausea, and others (Ministerio de Salud de Perú, 2024). The exclusion criterion considered was incomplete data recording.

### 2.4 Samples

Blood serum samples from AFI patients were collected in Vacuette Serum Separator Clot Activator tubes (Vacuette, Greiner Bio-One, Kremsmünster, Austria). The serum was separated from blood at 5,000 RPM for 10 min at 4°C and subsequently stored at −80°C.

### 2.5 DENV and DENV-2 detection through real-time PCR

The viral RNA extraction was conducted from a 200 μl serum sample, following the protocol recommended by the manufacturer of the High Pure Viral RNA kit (Roche Applied Science, Mannheim, Germany). DENV detection was achieved through a real-time RT-PCR assay utilizing primers and probes designed by Leparc-Goffart et al. (2009; Supplementary Table S1). The reaction mixture was prepared according to the guidelines of the commercial LightCycler^®^ Multiplex RNA Virus Master kit (Roche Diagnostics, Deutschland-Mannheim, Germany), using 10 pmol/μL of primers and probes for a final volume of 20 μL. Amplification was programmed for 8 min at 55°C for reverse transcription, followed by 30 s at 95°C for pre-denaturation, 55 cycles of amplification at 95°C for one second for denaturation, 20 s at 55°C for annealing, 1 s at 72°C for extension, and finally 30 s at 40°C for cooling. For DENV-2 detection, primers and probes designed by Johnson et al. (2005; Supplementary Table S1) were employed under the same RT-PCR conditions as for DENV, with the annealing temperature set at 57°C. In each process, a positive sample and nuclease-free water were included as amplification and contamination controls.

### 2.6 Amplification and sequencing of envelope gene (E gene)

For complete E gene amplification, we used viral RNA from samples that tested positive for DENV-2 and had a high relative viral load with a CT value ≤ 20. The E gene amplification primers previously designed by Santiago et al. (2019; Supplementary Table S1) were utilized, and amplification was conducted in two steps. First, cDNA synthesis was performed with 50 pmol/μL of random hexamer primer, 600 pmol/μL of Anchored-oligo(dT)18 primer, 10 mM of dNTP, 40 U/μL of protector RNase Inhibitor, and 20 U/μL of Transcriptor Reverse Transcriptase included in the Transcriptor First Strand cDNA Synthesis kit (Roche Diagnostics, Mannheim, Germany), combined with 5 μL of RNA for a final mixture of 20 μL for reverse transcription (RT). Reverse transcription was conducted at 65°C for 10 min of denaturation and 60 min of retrotranscription at 50°C. Second, E gene amplification was conducted using the master mix from the LightCycler FastStart DNA Master HybProbe kit (Roche Diagnostics, Deutschland-Mannheim, Germany) for a final PCR volume of 40 μL. Amplification conditions were set at 95°C for 10 min of pre-denaturation, followed by 55 amplification cycles at 94°C for denaturation for 1 min, 59°C for 1 min for annealing, and 72°C for 2 min of extension, followed by a final extension at 72°C for 10 min in a PCR Mastercycler Nexus GX2, Eppendorf thermocycler.

The amplification products were observed on a 1.2% agarose gel using the fluorescent DNA dye SafeView™ Classic (Applied Biological Materials Inc, Canada) in a UV transilluminator equipped with a photo documentation system in MUV26 gel (Major Science, USA-California). This setup facilitated the determination of amplicon sizes of 1,525 bp. PCR amplicon purification followed the protocol of the SprinPrep™ Gel DNA kit (Novagen, USA), ensuring that only amplicons with strong band intensity were purified. Finally, the purified PCR products were sequenced, and measurements using a NanoDrop™ One (Thermo Scientific™, USA) spectrophotometer indicated a concentration of ~30–60 ng/μl and an optimal quality ratio (260 nm/280 nm) of 1.8–2.0. Sequencing was performed using the Sanger method with primer sets for the DENV-2 E gene designed by Johnson et al. (2005; Supplementary Table S1) in an automated capillary electrophoresis sequencer ABI 3730xl by Macrogen (Macrogen, Inc. South Korea).

### 2.7 Analysis of sequences and data sets

The review of electropherograms, along with the editing and assembly of the nucleotide sequences generated by this study, was conducted using the CodonCode Aligner version 10.0.2 software (CodonCode Corporation, Dedham, MA).

A dataset consisting of public nucleotide sequences of the E gene, downloaded from the National Center for Biotechnology Information (NCBI)'s GenBank database up to October 2023, was prepared. This dataset, along with the sequences obtained in this study, served as a reference for the required analyses. The data group was reduced to 78 sequences that represent the entire genotypic and intragenotypic diversity known for DENV-2 in Peru (Supplementary Table S2).

Previously, the diversity of Peruvian strains was analyzed in a global context using the Neststrain platform, developed for real-time monitoring of the evolution of the DENV virus (Hadfield et al., 2018). To this end, 3,713 DENV-2 E gene sequences from around the world, deposited in the GenBank database and sampled until December 2022, were examined. The sequences obtained in this study were incorporated into the global analysis using the Nextclade v3.10.2 tool, and a maximum likelihood phylogeny with a time scale was reconstructed. In addition, the frequency of DENV-2 genotypes in Peru was evaluated over time. Following this preliminary analysis, repetitive sequences and non-Peruvian sequences that were not related to Peruvian diversity were excluded because they did not group together or present an immediate phylogenetic relationship with the sequences of Peruvian origin. Sequences were labeled with the accession number/country/isolation year format.

### 2.8 Phylogenetic analysis

The multiple alignments of the reference dataset with the sequences obtained in this study were conducted on the MEGA v11.0 platform using the MUSCLE algorithm (Tamura et al., 2021). Alignments of 1,485 bp, corresponding to the size of the DENV-2 E gene, were obtained. The nucleotide substitution model that best represents the phylogenetics of our datasets was determined using the jModelTest 2.1.10 program (Darriba et al., 2012).

The reference data set (*n* = 78), which includes all representative DENV-2 sequences reported in Peru along with the sequences obtained in this study, comprised a 110-sequence alignment. This alignment revealed that the TIM2 + G + I model was the optimal nucleotide substitution model according to the Akaike Information Criterion (AIC) and the Bayesian Information Criterion (BIC), and it was used in the E gene phylogenetic reconstruction.

The reconstruction of DENV-2's phylogenetic tree was conducted using the Maximum Likelihood (ML) method with 1,000 bootstrap repetitions, supplemented by the approximate Bayes test (aBayes) as a single branch test through the IQ-TREE 2.2.0 program (Minh et al., 2020). Branches with bootstrap values ≥ 70 and posterior probabilities (PP) ≥ 0.7 were considered reliable. The consensus phylogenetic trees obtained were visualized using FigTree v1.4.4.

Due to the lack of consensus on the intraserotypic classification of the cosmopolitan genotype, previously described whole-genome and E-gene-based phylogenetic analyses (Phadungsombat et al., 2018; Suzuki et al., 2019; Yenamandra et al., 2021) were used as references.

### 2.9 Distance and identity matrices

The mean genetic distances among the complete alignments of the sequences obtained in this study, their immediate ancestors, and their oldest ancestors—based on their phylogenetic relationships within each genotype—were estimated to assess the degree of divergence achieved over time. The estimation of evolutionary divergence between pairs of sequences from different groups was performed using the DISTANCE tool from the MEGA v11.0 platform. The p-distance method (number of nucleotide differences/total sequence length) was used to calculate the mean genetic distances, modeling the variation in the rate across sites with a 4-parameter gamma distribution. The variance was estimated via the bootstrap method with 1,000 repetitions. Following the configuration processing, a distance matrix (p-distance) was derived to evaluate the genetic divergence between the analyzed groups. Additionally, the identity percentages at both nucleotide and amino acid levels were calculated to evaluate the level of conservation and variation among the analyzed sequences.

### 2.10 Analysis of mutations in the E protein sequence

The translation of nucleotide sequences into amino acids was conducted in MEGA v11.0. To study the non-synonymous changes in the sequence of 495 amino acids of the DENV-2 envelope (E) protein, the oldest prototype strain JAM_95_83 (DQ364484; Jamaica 1983) was used as a reference for the Am/As genotype, and the oldest strain DENV-2/ID/1023DN/1975 (GQ398263; Indonesia 1975) was used for the cosmopolitan genotype. In both cases, reference sequences were included to position the sequences of this study within the lineages and sub-lineages to which they belong based on their determined phylogenetic relationships.

### 2.11 Structural analysis of mutations and mapping to antigenic sites

For the structural analysis of the mutations, the complete crystal structure of the mature DENV-2 envelope (PDB:3J27), retrieved from the Protein Data Bank (RCSB PDB, http://www.pdb.org), served as a template. The E protein modeling for each variant was conducted using the homology method in ColabFold v1.5.5 software. Five intermediate models were generated for each modeling attempt, and in each case, the model with the best scores was chosen. Structural alignments were performed using Swiss-PdbViewer v4.1 software. Non-synonymous mutations identified in each variant were mapped to the antigenic determinants previously described for the DENV-2 envelope (Supplementary Table S3; Sarker et al., 2023). The three-dimensional structure and folding of the E protein were analyzed with PyMol v3.0 software (http://www.pymol.org). Visualization, editing, and annotating of new mutations were executed using UCSF Chimera v1.16 software (https://www.cgl.ucsf.edu/chimera/).

### 2.12 Changes in DENV-2 diversity during outbreaks

The temporal segregation of DENV-2 diversity in Cajamarca was conducted during the study period, categorizing the different variants according to the year of their identification. This aimed to analyze the relationship between changes in DENV-2 intraserotypic diversity and the emergence of DENV outbreaks identified in this study. The outbreaks were identified using real-time RT-PCR and sequencing techniques. Additionally, data on DENV incidence in Cajamarca, reported by CDC Peru-MINSA during the sampling period, were analyzed.

## 3 Results

### 3.1 DENV outbreaks in the region of study

**Figure 1:**
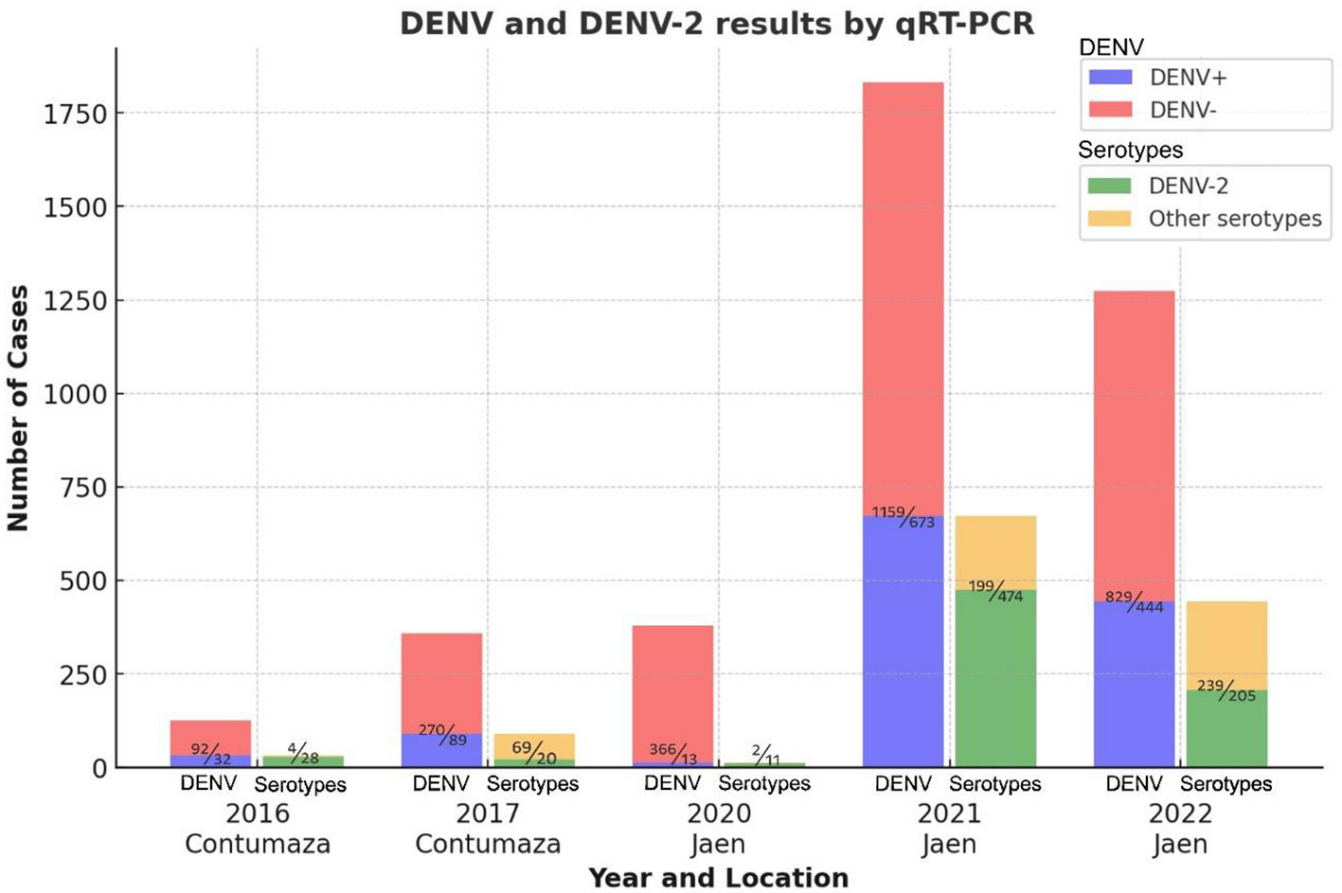
DENV and serotyping results by year and location of sampling. The bar graph shows DENV and serotyping results by sampling year and location. DENV+ indicates positive, DENV- indicates negative for dengue, and other serotypes include all DENV serotypes except DENV-2. Numbers above the colored bars, separated by a diagonal line, indicate the number of cases as appropriate.

A total of 3,967 blood serum samples from patients with AFI were analyzed using the real-time RT-PCR method to detect DENV. The number of positive and negative cases per sampling site and year is shown in Figure 1. In all cases, except for 2020, a positivity rate equal to or higher than 25% was observed. As shown in Table 1, the outbreaks under study were primarily associated with DENV-2 or exhibited a significant prevalence of DENV-2 exceeding 20%, as observed in 2017. The highest prevalence of DENV-2 was observed in 2016 at 87.50%.

**Table 1:** Frequency of DENV-2 cases in the study populations.

**Region**	**Province**	**Year**	**Sampling period**	**Collected samples (n)**	**DENV cases n (%)**	**DENV-2 cases n (%)**
Cajamarca	Contumaza	2016	January-June	124	32 (25.81)	28 (87.50)
Cajamarca	Contumaza	2017	January-June	359	89 (24.79)	20 (22.47)
Cajamarca	Jaen	2020	June-December	379	13 (4.43)	11 (84.62)
Cajamarca	Jaen	2021	January-December	1,832	673 (36.74)	474 (70.43)
Cajamarca	Jaen	2022	January-December	1,273	444 (34.88)	205 (46.17)

### 3.2 Clinical-demographic characteristics

Regarding the clinical-demographic data, the mean age was 30.2 years. In patients with dengue presenting warning signs, the mean number of days with fever was 2.6, which is higher than that of the group without warning signs. Additionally, in this group, myalgia, polyarthralgia, retro-ocular pain, nausea, and vomiting were more frequently observed among the general symptoms. Severe abdominal pain, chest pain, persistent vomiting, and decreased diuresis were characteristic of patients with dengue exhibiting warning signs. The clinical presentation regarding general symptoms was similar across both genotypes. In the Cosmopolitan genotype, dengue with warning signs was more prevalent (Table 2).

**Table 2:** Clinical-demographic characteristics based on the modified classification of dengue severity and DENV-2 genotypes.

**Clinical features**	**Dengue cases analyzed (*n* = 32)**	**Dengue without warning signs (*n* = 24)**	**Dengue with warning signs (*n* = 8)**	**Genotype of DENV-2: Am/As II (*n* = 8)**	**Genotype of DENV-2: Cosmopolitan (*n* = 24)**
Age in years	30.2 (SD: 13.6)	30.4 (SD: 13.8)	30.9 (SD: 14.1)	34.0 (SD:16.7)	29.5 (SD:11.9)
Male	13 (40.6%)	9 (37.5%)	4 (50.0%)	3 (37.5%)	10 (41.7%)
Fever	32 (100.0%)	24 (100%)	8 (100%)	8 (100%)	24 (100%)
Temperature at sample collection	38.5 (SD: 0.5)	38.5 (SD: 0.5)	38.6(SD: 0.4)	38.2 (SD :0.3)	38.6 (SD:0.6)
Duration of fever (days)	2.0 (SD: 1.6)	1.9 (SD: 1.5)	2.6 (SD: 2.5)	2.6 (SD: 2.4)	2.0 (SD: 1.6)
Arthralgia	28 (87.5%)	20 (83.3%)	8 (100%)	7 (87.5%)	21 (87.5%)
Hands polyarthralgia	16 (50.0%)	11 (45.9%)	5 (62.5%)	3 (37.5%)	13 (53.6%)
Feet polyarthralgia	15 (46.9%)	10 (41.7%)	5 (62.5%)	2 (25.0%)	13 (53.6%)
Arthritis	2 (6.6%)	1 (4.7%)	1 (12.5%)	1 (12.5%)	1 (4.7%)
Myalgias	27 (84.4%)	19 (79.1%)	8 (100%)	5 (62.5%)	22 (92.2%)
Headache	28 (87.5%)	21 (87.5%)	7 (87.5%)	7 (87.5%)	21 (87.5%)
Ocular or retroocular pain	20 (62.5%)	14 (58.3%)	6 (75.0%)	4 (50.0%)	16 (67.7%)
Lumbar pain	12 (37.5%)	8 (33.3%)	4 (50.0%)	3 (37.5%)	9 (38.0%)
Skin rash	3 (9.4%)	1 (4.7%)	2 (25.0%)	2 (25.0%)	1 (4.7%)
Conjunctival injection	1 (3.1%)	0 (0%)	1 (12.5%)	0 (0%)	1 (4.7%)
Non-purulent conjunctivitis	3 (9.4%)	2 (8.3%)	1 (12.5%)	1 (4.7%)	2 (8.3%)
Throat pain	4 (12.5%)	1 (4.7%)	3 (37.5%)	2 (25.0%)	2 (8.3%)
Lack of appetite	1 (3.1%)	1 (4.7%)	0 (0%)	0 (0%)	1 (4.7%)
Nausea	12 (37.5%)	7 (29.1%)	5 (62.5%)	3 (9.4%)	9 (37.5%)
Vomiting	12 (37.5%)	7 (29.1%)	5 (62.5%)	3 (9.4%)	9 (37.5%)
Severe abdominal pain	6 (18.6%)	0 (0%)	6 (75.0%)	2 (25.0%)	4 (16.6%)
Chest pain or dyspnea	3 (9.4%)	0 (0%)	3 (37.5%)	2 (25.0%)	3 (13.0%)
Persistent vomiting	1 (3.1%)	0 (0%)	1 (12.5%)	0 (0%)	1 (4.7%)
Diuresis decreases	1 (3.1%)	0 (0%)	1 (12.5%)	0 (0%)	1 (4.7%)
Hepatomegaly	1 (3.1%)	1 (4.7%)	0 (0%)	0 (0%)	1 (4.7%)

SD, Standard deviation.

### 3.3 Determination of nucleotide sequences of the DENV-2 E gene

A total of 32 complete sequences of the DENV-2 E gene (1,485 bp) were successfully obtained; eight corresponded to the American/Asian (Am/As) genotype, while 24 belonged to the cosmopolitan genotype according to preliminary analysis (Supplementary Figure S1). At least two sequences were obtained per outbreak or year of sampling, and all E gene sequences from this study were stored in the GenBank database, available under the accession codes mentioned in Table 3.

**Table 3:** Sequences obtained in this study.

**N°**	**Province**	**Isolation date**	**Sequence name**	**Accession number**	**Genotype**
1	Contumaza	06/2016	PeCa21.06_2016	PP732432	Am/As II
2	Contumaza	06/2016	PeCa22.06_2016	PP732433	Am/As II
3	Contumaza	05/2017	PeCa17.05_2017	PP732428	Am/As II
4	Contumaza	04/2017	PeCa18.04_2017	PP732429	Am/As II
5	Contumaza	05/2017	PeCa19.05_2017	PP732430	Am/As II
6	Contumaza	03/2017	PeCa20.03_2017	PP732431	Am/As II
7	Jaen	11/2020	PeCa2.11_2020	PP732423	Cosmopolitan
8	Jaen	10/2020	PeCa9.10_2020	PP732426	Am/As II
9	Jaen	11/2020	PeCa13.11_2020	PP732427	Am/As II
10	Jaen	04/2021	PeCa1.04_2021	PP732409	Cosmopolitan
11	Jaen	10/2021	PeCa3.10_2021	PP732410	Cosmopolitan
12	Jaen	10/2021	PeCa4.10_2021	PP732412	Cosmopolitan
13	Jaen	06/2021	PeCa5.06_2021	PP732414	Cosmopolitan
14	Jaen	09/2021	PeCa6.09_2021	PP732415	Cosmopolitan
15	Jaen	06/2021	PeCa7.06_2021	PP732416	Cosmopolitan
16	Jaen	04/2021	PeCa8.04_2021	PP732417	Cosmopolitan
17	Jaen	07/2021	PeCa10.07_2021	PP732418	Cosmopolitan
18	Jaen	02/2021	PeCa11.02_2021	PP732419	Cosmopolitan
19	Jaen	11/2021	PeCa12.11_2021	PP732411	Cosmopolitan
20	Jaen	09/2021	PeCa14.09_2021	PP732420	Cosmopolitan
21	Jaen	01/2021	PeCa15.01_2021	PP732421	Cosmopolitan
22	Jaen	10/2021	PeCa16.10_2021	PP732422	Cosmopolitan
23	Jaen	11/2021	PeCa23.11_2021	PP732413	Cosmopolitan
24	Jaen	06/2022	PeCa24.06_2022	PP732400	Cosmopolitan
25	Jaen	08/2022	PeCa25.08_2022	PP732401	Cosmopolitan
26	Jaen	03/2022	PeCa26.03_2022	PP732402	Cosmopolitan
27	Jaen	02/2022	PeCa27.02_2022	PP732403	Cosmopolitan
28	Jaen	04/2022	PeCa28.04_2022	PP732404	Cosmopolitan
29	Jaen	07/2022	PeCa29.07_2022	PP732405	Cosmopolitan
30	Jaen	12/2022	PeCa30.12_2022	PP732406	Cosmopolitan
31	Jaen	12/2022	PeCa31.12_2022	PP732407	Cosmopolitan
32	Jaen	11/2022	PeCa32.11_2022	PP732408	Cosmopolitan

### 3.4 Phylogenetic analysis

**Figure 2:**
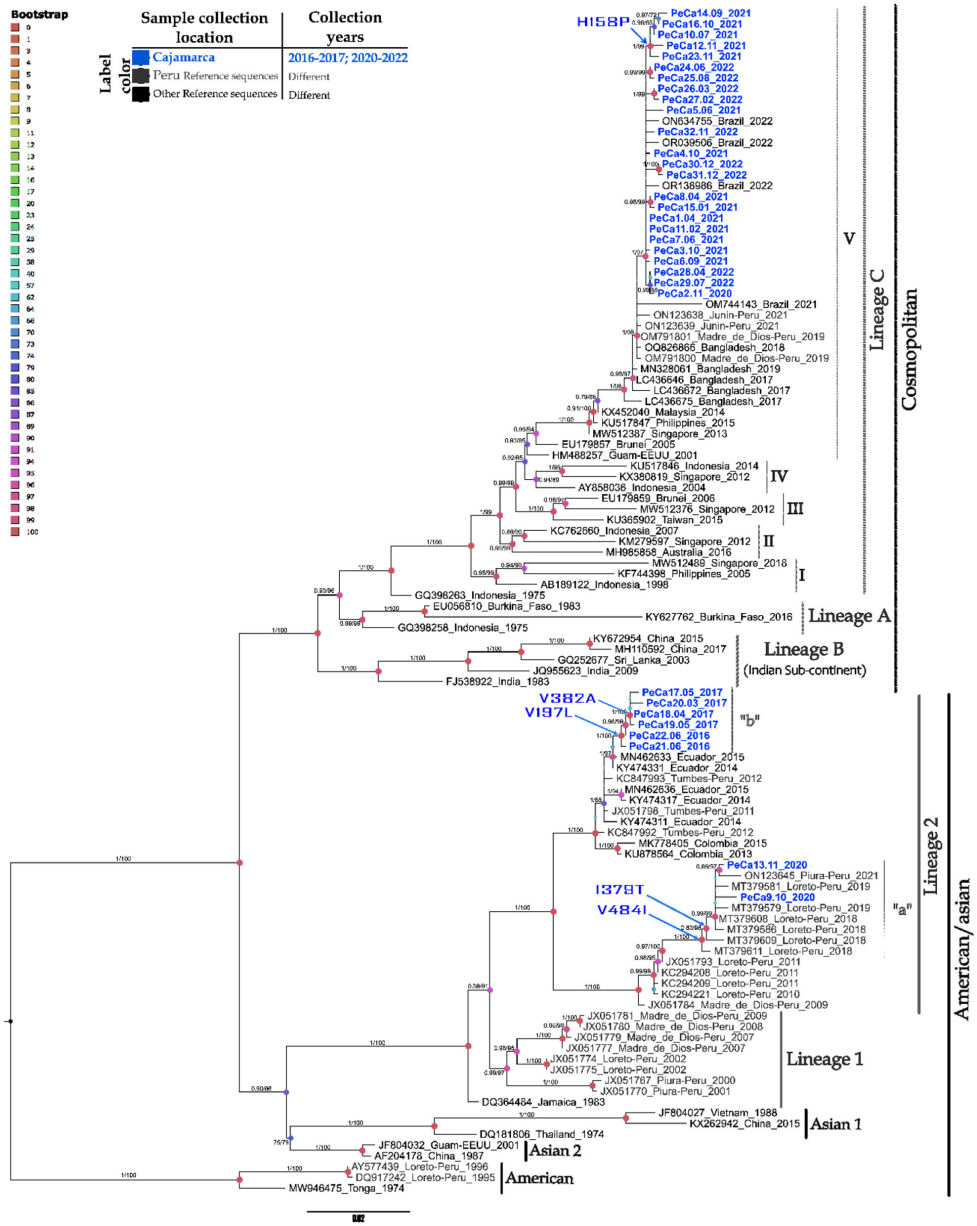
Phylogenetic tree of the DENV-2 E gene based on maximum likelihood. Phylogenetic tree of 110 complete sequences of the DENV-2 E gene. Prepared in IQTREE 2.0 with 1,000 bootstrap replications, approximate Bayesian computation, and the TIM2 + G + I model. Statistical significance values are represented by a color gradient (upper left corner), node size, and branch values. Vertical black bars group different genotypes, while gray bars group lineages within each genotype. Arrows on the nodes indicate amino acid changes at specific positions. This figure displays only support values ≥ 70 on the branches. Refer to the caption for the year and origin of the sequences. The scale at the bottom indicates the number of nucleotide substitutions per site across the branch.

The maximum likelihood (ML) phylogenetic analysis of 110 E gene sequences provided a phylogenetic tree with five large clades, each corresponding to one of the five genotypes that cause diseases in humans (Figure 2).

The DENV-2 strains in this study were divided into two major genotypes and compared with representatives of the Am/As and cosmopolitan genotypes. Within each genotypic group, clusters corresponding to the lineages that reflect their intragenotypic diversity were also formed. In the Am/As genotype, two lineages, lineage 1 and lineage 2, present in Peru, were identified. The Peruvian Am/As strains in this study showed affiliation solely with lineage 2 and formed diverse small clades within the lineage, which were supported by bootstrap values with upper branch confidence exceeding 90% and approximate posterior probabilities above 0.9 (Figure 2).

The Am/As genotype lineage (Am/As II) circulating in Peru showed a bifurcation into two larger monophyletic clades, with ancestral strains corresponding to isolates JX051784_FMD2285/PER/09 and JX051798_MIS1313/PER/11, which originated from the Madre de Dios and Tumbes regions, respectively. The percentage of sequence identity, the genetic distance between founder strains, geographical location, and temporal distance suggest that this lineage had a dual origin during its introduction into Peruvian territory (Table 4, Supplementary Table S4).

**Table 4:** Genetic divergence among DENV-2 E Am/As II gene sequence groups.

**Sequence group**	**Year**		**[1]**	**[2]**	**[3]**	**[4]**	**[5]**	**[6]**	**[7]**	***[8]***	***[9]***
DQ364484/Jamaica	1983	[1]		0.0042	0.0039	0.0040	0.0041	0.0049	*0.0048*	*0.0049*	*0.0050*
^*^JX051784/Madre de Dios-Peru	2009	[2]	0.0263		0.0037	0.0039	0.0040	0.0029	*0.0026*	*0.0029*	*0.0032*
^*^JX051798/Tumbes-Peru	2011	[3]	0.0222	0.0215		0.0016	0.0018	0.0045	*0.0043*	*0.0044*	*0.0045*
Cajamarca *Am/As II “b”*	2016	[4]	0.0249	0.0242	0.0040		0.0009	0.0046	*0.0044*	*0.0045*	*0.0047*
Cajamarca *Am/As II “b”*	2017	[5]	0.0266	0.0259	0.0057	0.0024		0.0047	*0.0045*	*0.0046*	*0.0048*
Cajamarca *Am/As II “a”*	2020	[6]	0.0394	0.0152	0.0340	0.0367	0.0384		*0.0011*	*0.0012*	*0.0017*
^**^Loreto Am/As II “a”	*2018*	*[7]*	*0.0372*	*0.0130*	*0.0318*	*0.0345*	*0.0362*	*0.0045*		*0.0011*	*0.0017*
^**^Loreto Am/As II “a”	*2019*	*[8]*	*0.0384*	*0.0148*	*0.0330*	*0.0357*	*0.0374*	*0.0044*	*0.0042*		*0.0017*
^**^Piura Am/As II “a”	*2021*	*[9]*	*0.0397*	*0.0168*	*0.0343*	*0.0370*	*0.0387*	*0.0057*	*0.0062*	*0.0054*	

Evolutionary divergence was estimated using the p-distance method (the number of nucleotide differences/total sequence length), incorporating a 4-parameter gamma distribution and 1,000 bootstrap replicates to calculate the standard deviation in MEGA.

* Ancestral sequences of the Am/As II genotype introduced in Peru.

** A group of sequences of the Am/As II variant a that were not isolated in this study.

Cajamarca: a collection of sequences isolated in this region and categorized as indicated.

Values below the diagonal line represent the number of base differences per site.

Values above the diagonal line indicate the estimated standard error.

Each of the larger Am/As II lineage clades showed marked internal diversification made up of smaller clades that refer to lineage variants that arose from each founder strain over time. The ancestral variant from Madre de Dios gave rise to the so-called autochthonous variant Am/As II “a,” and the founder strain from Tumbes resulted in the so-called variant Am/As II “b.”

Variant Am/As II “a” comprised sequences obtained in the study region in 2020, integrated with sequences previously reported in Loreto between 2018 and 2019 and in Piura in 2021. Together, they shared a nucleotide identity between 98.40 and 98.65% regarding their ancestral variant (Supplementary Table S4). Variant Am/As II “b” consisted of sequences obtained in the Cajamarca region between 2016 and 2017; sequences detected in 2016 showed a 99.5% identity regarding their ancestral variant (Supplementary Table S4), while those detected in 2017 showed a close to 99.38% identity. It was evident that variant Am/As II “a” accumulated a larger number of nucleotide changes throughout the E gene compared to its closest ancestral variant.

The cosmopolitan genotype was divided into three major lineages (A, B, and C), and the cosmopolitan sequences from the northern Peruvian outbreaks were clustered within the same clade as the Peruvian, Brazilian, and Asian strains. These strains collectively belong to lineage C and sublineage V (C.V).

The cosmopolitan strain detected in Peru showed a close ancestral bond with a group of strains reported in Bangladesh between 2017 and 2019 (Figure 2, Supplementary Figure S1d). These strains are identified as their closest ancestors, forming a monophyletic group that delineates the geographical origin of the Peruvian cosmopolitan variant. The Peruvian cosmopolitan sequences were grouped within sub-lineage C.V, forming their own subclade that separates from their closest Bangladeshi ancestors with a statistical support of 98% branch confidence, an approximate PP of 1, and a nucleotide identity of 99.55% (Supplementary Table S5).

The Peruvian sub-lineage C.V. is derived from the isolated strain OM791801 PE/DB275/2019, taken from the Madre de Dios region. This sub-lineage includes strains identified in the Cajamarca region from 2020 to 2022, alongside those previously reported in Madre de Dios in 2019 and Junín in 2021. All strains in Cajamarca formed their own branch, distinct from others, with a bootstrap support of 97% and an approximate posterior probability (PP) of 1. Among the Cajamarca strains detected in 2021, a small group of sequences exhibiting divergence was identified, achieving a confidence level of 99% and an approximate PP of 1.

Phylodynamic analysis of the strains detected in this study (Supplementary Figure S1a) revealed that the reported Am/As II variants have a local Peruvian origin. As shown in Supplementary Figure S1b, the Am/As II “a” variant emerged in the Loreto region in 2018 and had already been detected in the Cajamarca region by 2019. Regarding the Am/As II “b” variant, a close phylogenetic link was identified with strains reported in Ecuador between 2014 and 2015, although they do not share all the substitutions observed in the Cajamarca strains (Supplementary Figure S1c). The time-scale phylogeny, along with the sequence similarity, indicated that the strain X051798_MIS1313/PER/11 is the closest known Peruvian ancestor of the Am/As II “b” variant. Conversely, the oldest Peruvian strain of the cosmopolitan genotype (OM791801_PE/DB275/2019) demonstrated ancestry solely with strains originating in Bangladesh, and the strains identified in Cajamarca were shown to be descendants of the first strain introduced in Peru (Supplementary Figure S1d). Assessment of genotype frequencies indicated that since 2020, the emerging cosmopolitan genotype has been co-circulating with increasing dominance over the native Am/As genotype in Peru and South America (Supplementary Figure S1e).

### 3.5 Genetic distances

The identified variants Am/As II “a” and Am/As II “b” exhibited high similarity to their respective ancestral strains JX051784/Madre_de_Dios, Peru, and JX051798/Tumbes, Peru. However, they demonstrated a sufficient level of divergence to be classified as variants within their lineage. Among the variants, the p-distances between Am/As II “a” and Am/As II “b” allowed each to cluster and form a genetically independent branch distant from the other. The Am/As II “a” variant was highly divergent from the Am/As II “b” variant, with distances ranging from 0.0345 to 0.0387. The ancestral strains also displayed significant divergence from one another, with a distance of 0.0215 at the base, reaching a distance of 0.0387 between their more distant branches. These divergence values are consistent with the dual Peruvian origin of the Am/As II lineage. The divergence values between strains of the same variant detected in different years are negligible. The distance matrix is shown in Table 4.

Within the cosmopolitan sub-lineage C.V, Peruvian strains did not show significant genetic distances from the founder strain OM791801/Madre de Dios-Peru (0.0017–0.0037) and the closest ancestral Asian strain LC436672 (0.0037–0.0067). Furthermore, no substantial genetic distances were found between the sequence groups detected in this study across different years (Table 5).

**Table 5:** Genetic divergence among sequence groups of the DENV-2 E cosmopolitan gene.

**Sequence group**	**Year**		**[1]**	**[2]**	**[3]**	**[4]**	**[5]**	**[6]**	**[7]**	***[8]***	***[9]***
GQ398263/Indonesia	1975	[1]		0.0047	0.0045	0.0047	0.0046	0.0047	0.0046	0.0045	0.0046
LC436672/Bangladesh	2017	[2]	0.0357		0.0016	0.0020	0.0018	0.0020	0.0018	0.0017	0.0019
^*^OM791801/Madre de Dios-Peru	2019	[3]	0.0340	0.0037		0.0014	0.0010	0.0014	0.0011	0.0008	0.0010
PeCa2.11_2020/Cajamarca	2020	[4]	0.0364	0.0061	0.0030		0.0009	0.0013	0.0010	0.0015	0.0012
Cajamarca	2021	[5]	0.0358	0.0053	0.0024	0.0019		0.0009	0.0005	0.0012	0.0008
Cajamarca (H158P)	2021	[6]	0.0370	0.0067	0.0037	0.0034	0.0028		0.0010	0.0015	0.0012
Cajamarca	2022	[7]	0.0364	0.0061	0.0031	0.0025	0.0022	0.0034		0.0012	0.0009
^**^Junin	2021	*[8]*	0.0343	0.0047	0.0017	0.0040	0.0034	0.0047	0.0041		0.0012
^**^Brazil	2022	*[9]*	0.0365	0.0074	0.0044	0.0047	0.0041	0.0054	0.0048	0.0054	

Evolutionary divergence was estimated using the p-distance method (the number of nucleotide differences/total sequence length), along with a 4-parameter gamma distribution and 1,000 bootstrap replicates to calculate the standard deviation in MEGA.

* Ancestral sequences of a cosmopolitan genotype were introduced in Peru.

** A group of cosmopolitan sequences that were not isolated in this study.

Cajamarca: a collection of sequences isolated in this region and categorized as indicated.

Values below the diagonal line indicate the number of base differences per site.

Values above the diagonal line indicate the estimated standard error.

### 3.6 New mutations in the E protein sequence

**Figure 3:**
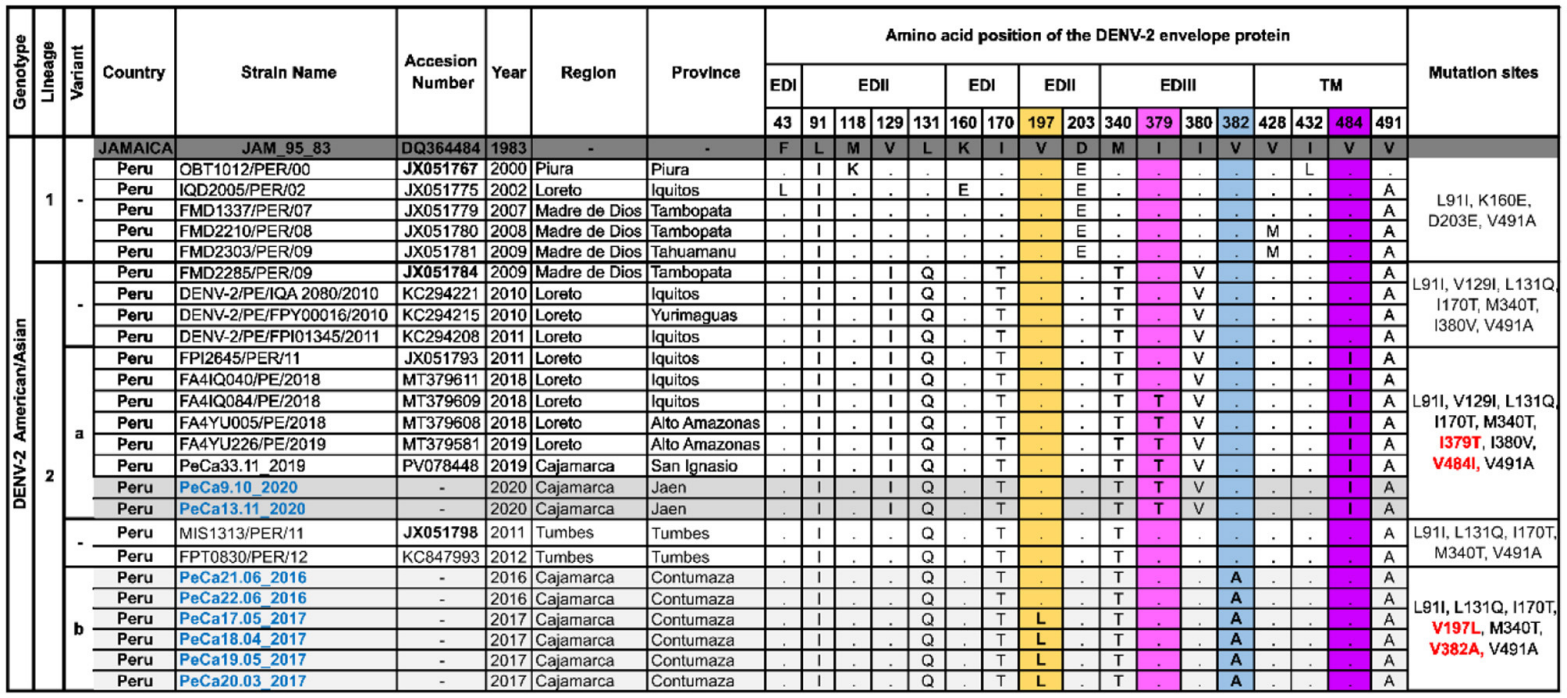
Polymorphisms of amino acids in the E protein of Am/As DENV-2. This figure shows the alignment of a set of amino acid sequences that are representative of the Peruvian Am/As genotype diversity and summarizes the specific sites where changes have been observed in more than one sequence compared to the DQ364484 prototype strain initially isolated in Jamaica. Letters indicate amino acid changes, while points signify site conservation. The column on the far right specifies the characteristic substitutions for each group of sequences. Novel mutations are highlighted in the colored columns. The nomenclature for new mutations is labeled in red, and the names of sequences obtained in this study are labeled in blue. The accessions of ancestral sequences for each variant are shown in bold.

The viral Am/As sequences characteristic of this study were compared to the prototype strain DQ364484_JAM_95_83 (Figure 3). Lineage 2 exhibited exclusive substitutions at positions 91, 129, 131, 170, 203, 340, and 380 of the envelope, distinguishing it from lineage 1, along with typical shared characteristics at positions 91 and 491. Ancestral strain MIS1313/PER/11 (JX051798) of the Peruvian lineage Am/As II differs from strain FMD2285/PER/09 (JX051784) by not showing changes at positions 129 and 380.

Unique and autochthonous mutations were identified in FMD2285/PER/09 (JX051784) descendants. The viruses detected in Cajamarca in 2020, along with the strains from Loreto reported between 2018 and 2019, which were grouped in the Am/As II “a” variant, exhibited single nucleotide polymorphisms at site 379 (I379T) of the EDII domain and at site 484 (V484I) of the transmembrane regions (Figure 3). The Am/As II “a” variant displayed an amino acid profile similar to that of the FPI2645/PER/11 (JX051793) strain, which was isolated during a dengue outbreak in Loreto in 2011, potentially explaining its origin.

The MIS1313/PER/11 (JX051798) descendants also developed native and novel mutations. Viruses detected in the Cajamarca region between 2016 and 2017 that comprise variant Am/As II “b” were characterized by non-synonymous substitutions at positions 197 (V197L) and 382 (V382A) of the viral envelope located in the EDII and EDIII domains, respectively (Figure 3). The sole presence of V382A in strains from 2016 and both substitutions in strains of variant Am/As II “b” from 2017 indicate that variants originate and accumulate mutations gradually (Figure 3, Supplementary Figure S1c). A similar observation was made with variant Am/As II “a,” where some strains isolated in Loreto in 2018 exhibited a unique polymorphism at position IV484I, just like its possible immediate predecessor FPI2645/PER/11 (JX051793), which was isolated in the same region in 2011 (Figure 3).

**Figure 4:**
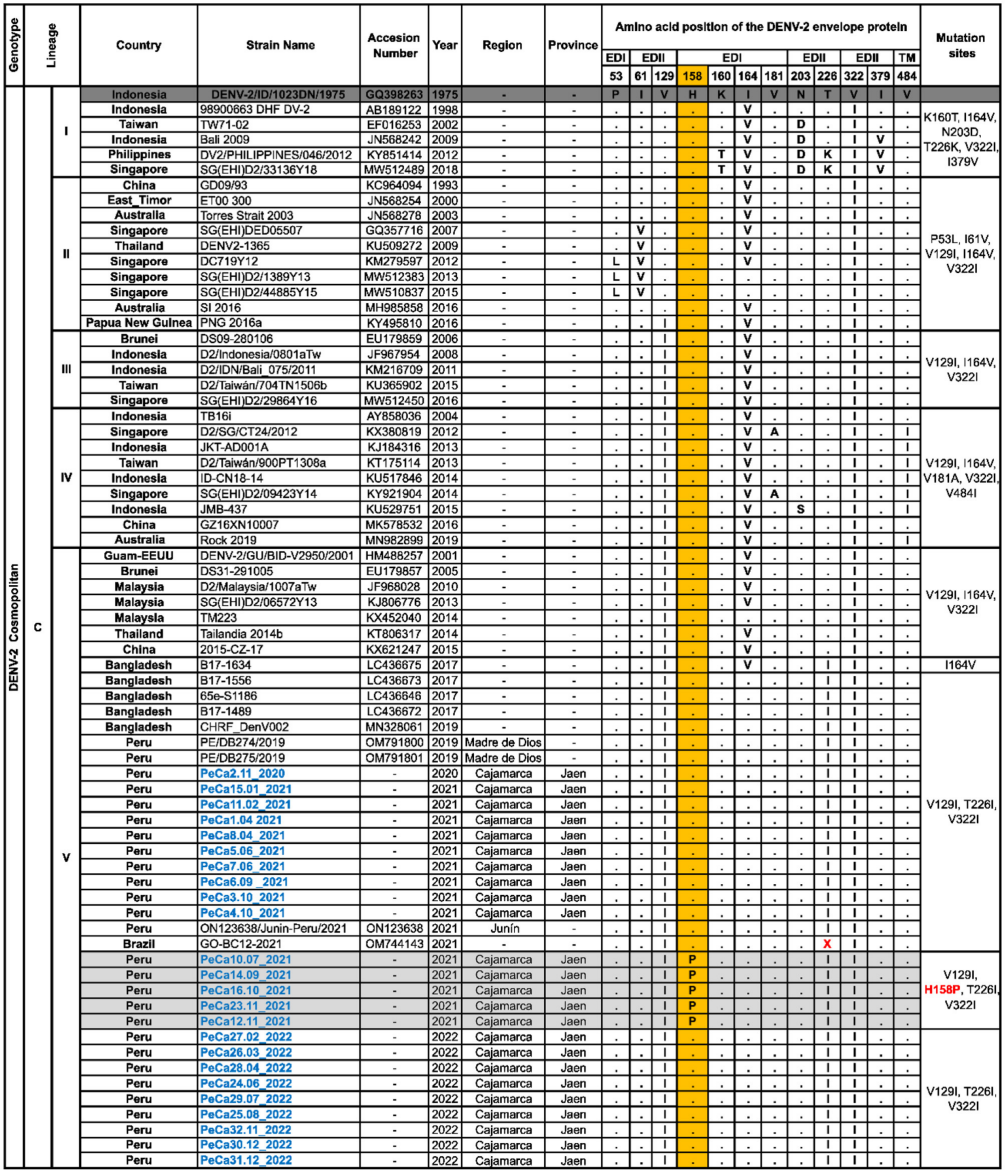
Polymorphisms of amino acids in the E protein of cosmopolitan DENV-2. This figure illustrates the alignment of a set of amino acid sequences that are representative of the cosmopolitan genotype diversity introduced in Peru. It summarizes the specific sites where changes have been observed in more than one sequence compared to the GQ398263 prototype strain, which was first isolated in Indonesia. Letters indicate changes in amino acids, while points indicate site conservation. The letter “X” signifies the absence of an amino acid. The column on the right specifies the characteristic substitutions for each group of sequences. Novel mutations are highlighted in the colored columns, with the nomenclature of new mutations labeled in red and the names of the sequences obtained in this study labeled in blue.

The sequences of cosmopolitan viral strains described in this study for the first time were compared to the prototype strain GQ398263_DENV-2/ID/1023DN/1975. Within lineage C, notable substitution characteristics of each sub-lineage were observed, as shown in Figure 4. Particularly, sub-lineage C.V. displayed substitutions at positions 129, 164, 226, and 322. Additionally, it was noted that within sub-lineage C.V., substitution I164V was highly prevalent until 2017. However, a new variant has emerged since 2017, demonstrating a reversion in the mutation at position 164 and the acquisition of a new mutation, T226I.

The Peruvian cosmopolitan strains exhibited the same amino acid profile as the sub-lineage C.V. variant that emerged in Bangladesh in 2017. Most viral strains reported in this study, detected in the Cajamarca region between 2020 and 2022, did not show changes compared to the most ancestral strain OM791801_PE/DB275/2019, except for a small group of sequences obtained between July and November 2021, which revealed a new substitution in the EDI domain at position 158 (H158P) (Figure 4). Mutation H158P was no longer observed among the sequences analyzed in 2022.

### 3.7 Structural analysis of mutations and mapping to antigenic sites

**Figure 5:**
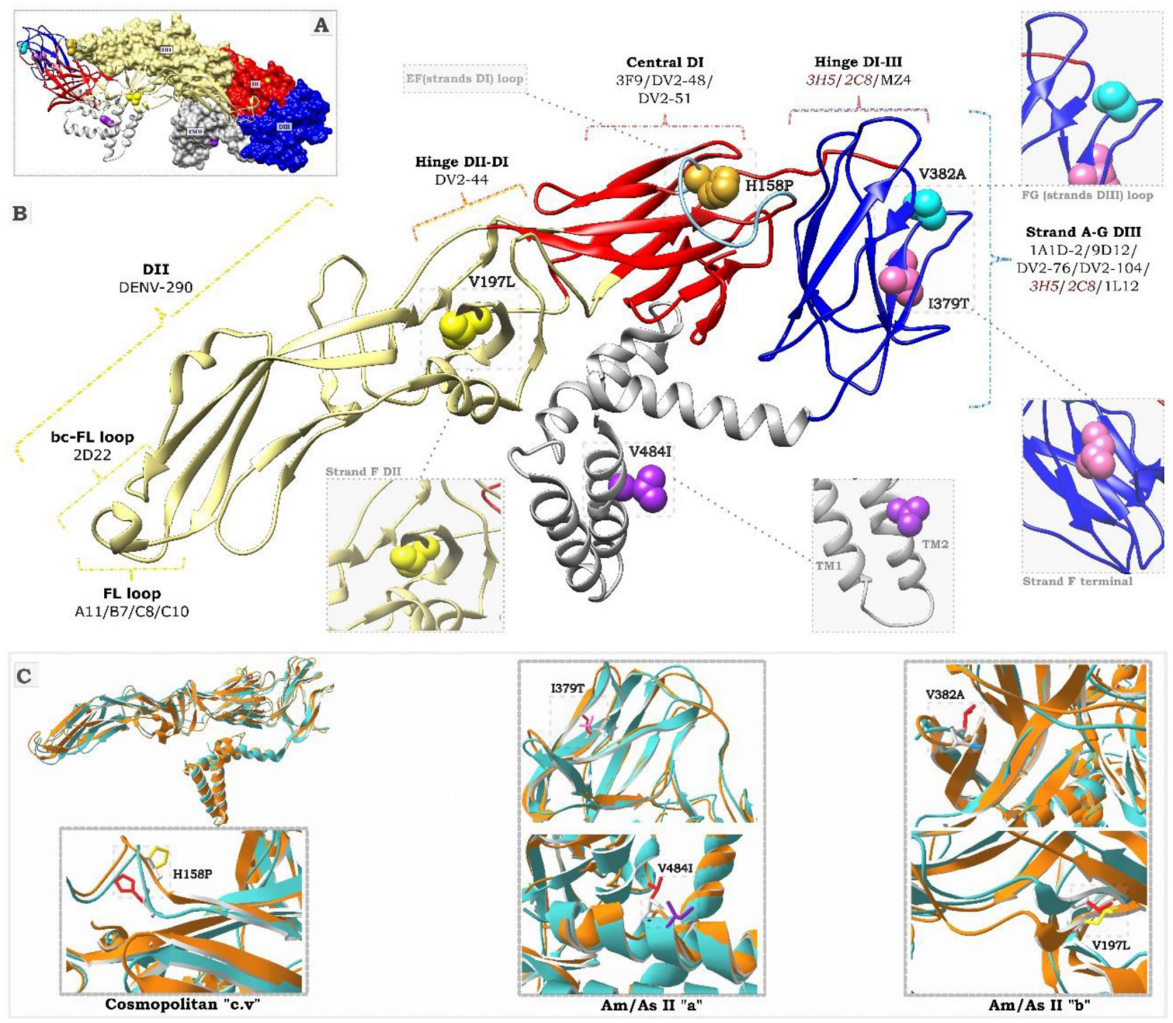
Structural representation of the DENV-2 E protein. **(A)** Shows the 3D dimeric structure of the mature E protein. DI, DII, DIII, and TM refer to domains I, II, III, and transmembrane of the protein. The colored residues correspond to the mutations described in this study. Substitutions I379T (pink) and V484I (purple) correspond to the Am/As II “a” variant. Substitutions V197L (yellow) and V382A (light blue) correspond to the Am/As II “b” variant. The H158P substitution for the cosmopolitan variant “cv” is highlighted in mustard. **(B)** Displays the 3D folding of the E protein monomer. The colored keys indicate DENV-2-specific monoclonal antibodies and the locations of their epitope regions (specific epitopes for each antibody are presented in Supplementary Table S3). Antibodies in red induce the ADE effect. The black dotted lines indicate an enlargement of the positions of the mutant residues. **(C)** Presents the alignment of the 3D structures of protein E. The cyan structures correspond to the template (PDB:3J27), while the orange structures represent the variants identified in this study, labeled with their characteristic substitutions.

Mutation mapping along the antigenic regions of the E protein (Figure 5A) revealed that the I379T and V382A substitutions are located in the antigenic site of EDIII, referred to as the lateral ridge (Figure 5B). Specifically, I379T was found in the terminal segment of the F chain, whereas V382A was located in the loop connecting the F and G strands, comprising residues 381–385. In the FG loop, the V382A mutation flanks the key residue E383, an epitope specific to monoclonal antibodies 1A1D-2, 9D12, 3H5, and 2C8, which exerts a specific neutralizing effect on DENV-2 (Supplementary Table S3). Furthermore, antibodies 3H5 and 2C8 also induce the antibody-dependent enhancement (ADE) phenomenon (Figure 5B; Supplementary Table S3). The V197L mutation was located in the F chain of the EDII domain, very close to the DI/DII hinge antigenic site (Figure 5B). The H158P mutation was located in the center of the EF loop of the EDI, which constitutes a conformational antigenic site of DENV-2 that specifically interacts with NAb 3F9. H158P is also part of the dimeric epitope comprising residues 148-159, specific to NAbs A11, B7, C8, and C10 (Supplementary Table S3). The V484I mutation was located in the second helix of the TM domain, outside the antigenic region of the E protein, but was essential for phylogenetic classification.

Structural predictions showed local modifications in the region of the protein affected by the mutations (Figure 5C). The structural alignment indicated a displacement of neighboring residues, which affected the local overlap with the template to varying degrees in all cases. In particular, the acquisition of an alanine residue, a smaller amino acid, at position 832 of the Am/As II “a” variant and a threonine, a polar residue, at position 379 of the Am/As II “b” variant altered the spatial orientation and folding of the FG loop of EDIII. Similarly, the substitution of a proline, an apolar residue, at position 158 of the cosmopolitan variant c.v. caused changes in the spatial orientation of the EF loop of EDII as well as in the side chain arrangement of the new amino acid. The substitution of valine, a small amino acid, with larger amino acids, such as isoleucine at position 484 and leucine at position 197, influenced the side chain arrangement of the new residue in the transmembrane (TM) region and the position of the F chain of the EDII domain, respectively.

### 3.8 Changes in DENV-2 diversity during outbreaks

**Figure 6:**
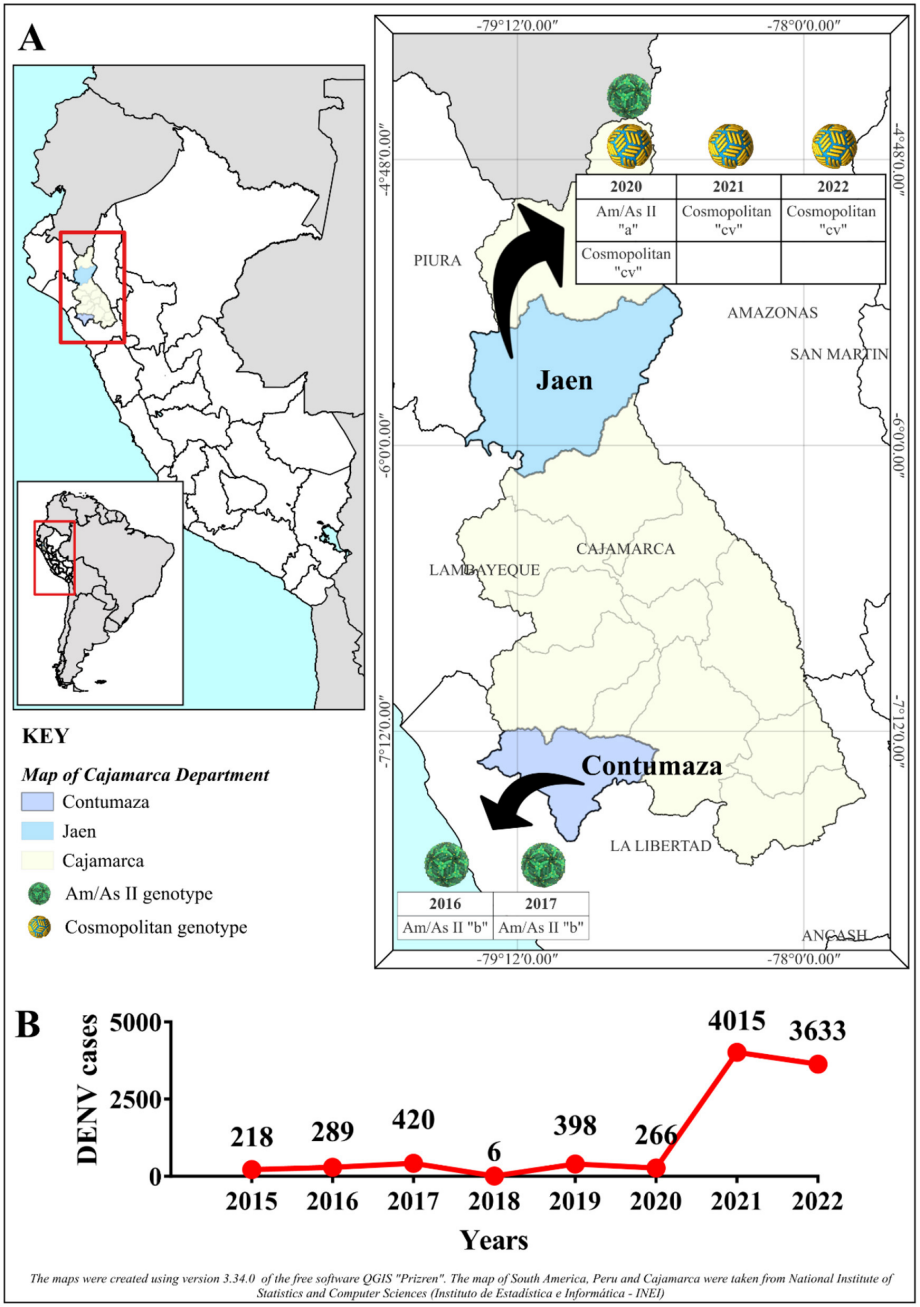
Intra-serotypic representation of DENV-2 in Cajamarca. **(A)** Illustrates the geographical location of the Cajamarca region (in yellow) and the provinces of Contumaza (in blue) and Jaén (in light blue), where the DENV density is concentrated during the indicated years. The temporal segregation of the intraserotypic diversity of DENV-2 is represented within the area's boundaries. The color of the spheres corresponds to different genotypes, as described in the caption, and for each sphere, the year and variant reported in this study are detailed. For the Am/As II (lineage 2) genotype, the letter “a” refers to the variant Am/As II “a,” while the letter “b” indicates the variant Am/As II “b.” In the case of the Cosmopolitan genotype, “cv” signifies sub-lineage “cv.” The black arrows denote the temporary segregation within the provinces. **(B)** Displays the annual incidence of DENV cases reported by the National Center for Epidemiology, Prevention, and Control of Diseases (CDC-Peru)—Ministry of Health (Ministerio de Salud, MINSA) in Cajamarca, highlighting the variation in DENV cases.

According to DENV incidence data collected by CDC Peru–MINSA (MINSA, 2023; Suzuki et al., 2019) during the study period (Figure 6B), endemic outbreaks took place in Cajamarca, with predominance or important presence of DENV-2, as shown in Table 1. These outbreaks were characterized by consecutive changes in the genotypic and intragenotypic composition of DENV-2 (Figure 6A).

DENV-2 genotypes and variants found in Cajamarca had a temporal distribution from 2016 to 2022. The endemic outbreak of 2016 was characterized by the presence of preceding strains of the Am/As II “b” variant, and 1 year later, the Am/As II “b” variant, which, as already described, had structurally consolidated, was found to be circulating during the 2017 outbreak. During the 2020 outbreak, a different variant of the Am/As II lineage, the Am/As II “a” variant, was identified. As shown in Figure 6B, no DENV outbreak occurred in Cajamarca in 2018, but it re-emerged in 2019. Furthermore, between 2018 and 2019, the Am/As II “a” variant was already present in the DENV outbreaks in the Loreto region (Figure 2, Supplementary Figure S1b), the same variant detected in Cajamarca in 2019 with the PeCa33.11_2019 strain (Accession number: PV078448).

During the 2020 outbreak, the cosmopolitan genotype variant introduced in Peru and the cocirculating Am/As II “a” variant were both present. However, between 2021 and 2022, only the cosmopolitan genotype was recorded. The year 2020 proved to be a transition period between the Am/As II genotype and the cosmopolitan one, with both genotypes-co-circulating in Cajamarca.

## 4 Discussion

DENV is a re-emerging etiological agent that has successfully spread to almost all regions of Peru. During the last decade, it has accounted for more than 50% of arbovirus infection cases in the Cajamarca region (MINSA, 2018; del Valle-Mendoza et al., 2020). Furthermore, three serotypes (DENV-1 to DENV-3) have been identified, with a predominance of DENV-2 in 2016 (MINSA, 2018; del Valle-Mendoza et al., 2020), 2018, and 2019 (INS, 2020), and DENV-3 in 2017 (Aguilar-Luis et al., 2021). Except for 2017, DENV-2 has been responsible for the recent DENV outbreaks in Cajamarca. This is the first study in which DENV serotype 2 has been molecularly characterized in the region, and the results reflect the situation in other endemic regions of Peru with regard to DENV molecular epidemiology.

The phylogenetic analysis in this study revealed that two of the five genotypes comprising DENV-2, the Am/As genotype and the cosmopolitan genotype, circulated in Cajamarca between 2016 and 2022 (Twiddy et al., 2002; Waman et al., 2016). The Am/As genotype was identified as circulating during the outbreaks of 2016 and 2017, while it was found that both genotypes co-circulated during the outbreak of 2020. However, the cosmopolitan genotype was the only circulating genotype identified during the outbreaks of 2021 and 2022, which may indicate a possible genotypic substitution event resulting in the displacement of the Am/As genotype. The identification of the Am/As 2II genotype aligns with findings from previous studies in Peru. Although documentation is scarce, this has been the most studied genotype in the Amazon regions (Mamani et al., 2011; Williams et al., 2014; Morrison et al., 2010). The introduction of Am/As I and II has been linked to the emergence of severe dengue epidemic outbreaks (Montoya et al., 2023; Cruz et al., 2013) and has been identified in neighboring countries such as Brazil (INS, 2020; Figueiredo et al., 2014), Colombia (de Jesus et al., 2020; Laiton-Donato et al., 2019; Méndez et al., 2012), and Ecuador (Real-Cotto et al., 2017). It is considered the prevailing genotype across all of the Americas and the Caribbean (Mir et al., 2014; Añez et al., 2011). Moreover, the identification of the cosmopolitan genotype in our study was highly likely, as it was recently reported as an imported case in Peru (García et al., 2022). However, our report provides the first evidence of the cosmopolitan genotype's expansion in northern Peru.

In Peru, recent studies reveal a rapid expansion of the cosmopolitan genotype of DENV-2 in regions such as Lambayeque, Lima, Cajamarca, Huánuco, Junín, Loreto, and Madre de Dios up until 2022. Similarly, the spread of DENV-1 genotype V is reported in Ancash, Lima, Cuzco, Cajamarca, and Ucayali. Both genotypes are circulating simultaneously across various regions of Peru (Bailon et al., 2025).

The presence of the cosmopolitan genotype in Cajamarca in 2020 was alarming due to the speed of its transmission since it was first reported in Peru and America in September 2019 in Madre de Dios (García et al., 2022), a region located in southeastern Peru at a significant geographical distance from Cajamarca and separated by the Andes as a geographical barrier. It is still unclear how long the cosmopolitan genotype has been circulating in Peru, especially considering that in Acre state, Brazil, which borders the Madre de Dios region, the first DENV-2 cosmopolitan cases were only reported in 2021 (Giovanetti et al., 2022; Amorim et al., 2023), 2 years later. This suggests that the cosmopolitan genotype may have begun circulating in Peru long before its first notification.

It has also been described that the Am/As sequences of the outbreaks under study were exclusively grouped with Am/As II sequences that were isolated in Peru in previous studies, demonstrating that they were autochthonous viral strains. Thus, the possibility that the outbreaks under study emerged due to the importation of new viral strains was ruled out. This research reports two Am/As II variants that could be related to the emergence and severity of each outbreak noted in this study since 2016. The Am/As II “a” variant, identified in Cajamarca in 2020, was previously reported as the cause of serious dengue outbreaks in Loreto between 2018 and 2019 (Falconi-Agapito et al., 2020) and likely spread to Cajamarca in 2019, causing the re-emergence of DENV in that region (Minh et al., 2020), similar to what occurred in Loreto. Moreover, the Am/As II “b” variant, reported for the first time in this study, could have originated in Cajamarca due to the presence of its precursor strains with a single substitution, which may indicate a local microevolutionary process. Both the Am/As II “a” and Am/As II “b” variants may have influenced viral transmissibility, possibly reflected in the occurrence of outbreaks and increased DENV prevalence during the years in which they were detected. Additionally, the emergence of new autochthonous viral variants or those introduced across borders has been identified as a primary factor in the emergence of highly clinically serious outbreaks (Falconi-Agapito et al., 2020; Dafalla et al., 2023; Kanan et al., 2024).

The molecular characterization of the amino acid sequence of the Am/AsII E revealed that the Am/As II “b” variant exhibited unique substitutions in the EDII and EDIII domains, whereas the Am/As II “a” variant displayed unique substitutions in the EDIII domain and the TM region, as previously described (Falconi-Agapito et al., 2020). The DENV envelope protein is involved not only in cell adhesion and membrane fusion but also serves as the primary target of the neutralizing immune response (Hu et al., 2021; Roehrig, 2003). Although the three domains (EDI, EDII, and EDIII) that comprise the ectodomain of the E protein possess antigenic potential, EDIII induces the production of the strongest neutralizing antibodies (NAbs; Fahimi et al., 2018; Poggianella et al., 2015), while EDII is also an important target of NAbs, albeit to a lesser extent.

The characteristic mutations of each described variant were located in antigenic sites involved in the neutralizing antibody response, the ADE phenomenon, and in sites that constitute epitopes recognized in vaccine design (Sarker et al., 2023). Previous studies have shown that changes in or near epitopes can affect antibody specificity against the E protein and configure an immune evasion or attenuation mechanism (Martinez et al., 2020; Samune et al., 2024). Therefore, the potential antigenic and immunogenic role of the new mutations cannot be ruled out.

We explored the potential impact of mutations on local structure, noting that non-conservative mutations, or conservative ones with a significant difference in the size of the replaced amino acid, could generate relevant changes in local structure, possibly affecting folding, affinity, or molecular recognition. Previous studies have shown that such mutations can modify the antigenicity of an epitopic site by reducing the binding affinity to neutralizing antibodies (NAbs; Wahala et al., 2010; Chen et al., 2022). Furthermore, since it has been reported that changes near the receptor binding sites (Roehrig et al., 2013) can affect viral interaction and infectivity strength (Hu et al., 2021; Kanan et al., 2024), our results raise the possibility that these mutations may contribute to changes in viral virulence. Although it is known that the E protein TM domain participates in dengue virus (DENV) assembly and expression, little is known about the effect of mutations on this domain. Some studies suggest that changes in amino acid composition or in the size of TM alpha helices may cause conformational changes that could be translated to the external domain of the E protein, altering its conformation and, therefore, the host's NAb specificity (Butrapet et al., 2011; Smith et al., 2011; Tsai et al., 2012). However, to confirm the impact of these modifications on protein stability and function, as well as their relationship with the pathogenicity and immune evasion of the virus, additional studies, such as molecular dynamics simulations and functional assays, are required.

This research characterized, for the first time, the cosmopolitan sub-lineage C.V that was introduced into South America and identified that substitutions V129I in EDII and V322I in EDIII were present in all C.V sequences. Regardless of their place of origin and year, these substitutions were also previously identified in some cosmopolitan strains (Suzuki et al., 2019), but their importance had not been described within lineage C's taxonomic classification. Likewise, all C.V sequences shared the substitution I164V (Ahmad et al., 2019; Kar et al., 2019) until the year 2017, when a new cosmopolitan variant, characterized by acquiring a substitution T226I in EDII and reverting site I164V to V164I in EDI compared to their immediate ancestral strains, emerged in Bangladesh. These novel mutations could be antigenic determinants contributing to their successful spread throughout South America (Giovanetti et al., 2022; Ciuoderis et al., 2023). The point mutation found in the Peruvian strains in Cajamarca in 2021 could be the first evidence of the beginning of the diversification of the Peruvian cosmopolitan strain; nevertheless, the absence of the H158P mutation in the strains detected in Cajamarca in 2022 might suggest that it was a temporary mutation that failed to establish itself in the population. Thus, conducting more studies in different Peruvian populations is necessary to verify its persistence or extinction.

Consecutive changes in the intraserotypic composition of DENV-2 occurred in Cajamarca, each accompanied by DENV outbreaks that gradually increased in magnitude and scope. Initially, these changes were marked by the replacement of the local Am/As II “b” and Am/As II “a” variants at the intragenotypic level between 2017 and 2019, which had a moderate impact on DENV prevalence. The absence of the Am/As II “b” variant in 2020 suggests its probable extinction, at least in Cajamarca, during 2018 and 2019. Although sampling was not conducted in 2019, the detection of the PeCa33_2019 strain in the region implies that the DENV outbreak in Cajamarca may have involved the introduction of the Am/As II “a” variant that year, which is native to the Loreto region. A second change in DENV-2 diversity in Cajamarca occurred with the introduction of a new variant of the cosmopolitan genotype c.v., of Bangladeshi ancestry, which replaced the Am/As II “a” genotype following a period of cocirculation around 2020. This change had a disproportionate impact on the prevalence of DENV toward 2021, exceeding the levels set by the introduction of the Am/As genotype (Montoya et al., 2023; Mamani et al., 2011). The replacement of DENV lineages (Laiton-Donato et al., 2019; Afreen et al., 2016), genotypes (Suzuki et al., 2019), and, to a lesser extent, serotypes, as observed in Cajamarca, is a common epidemiological phenomenon in highly endemic areas. The newly dominant viruses are more contagious (Yenamandra et al., 2021; Kotaki et al., 2016; Lin et al., 2021) and exhibit greater viral aptitude (Lee et al., 2012; Rodriguez-Roche et al., 2011) than those they replace. The rapid dispersion of the cosmopolitan genotype in Peru and South America (Amorim et al., 2023; Ciuoderis et al., 2023), which was reflected in a regional epidemic (PHAO, 2023), suggests an advantageous adaptive capacity and transmissibility compared to the Am/As genotype and other circulating serotypes.

During the 7 years of study, various external factors may have influenced the transmission dynamics of DENV-2 in Peru, potentially facilitating the displacement of the Am/As genotype by the cosmopolitan genotype. First, global climate changes and the events of the El Niño phenomenon (not a direct consequence of climate change), where sea surface temperatures (SST) rise by ≥ 0.4°C, have generated increases in temperature, precipitation, and humidity in the region, creating favorable conditions for the proliferation of the Aedes aegypti vector (Llanos-Cuentas and Altamirano-Quiroz, 2023). These conditions promote the formation of breeding sites, while the vector's expansion into higher altitude regions broadens its transmission area. Human migration at both national and international levels aids in the introduction and spread of the cosmopolitan genotype, while socioeconomic factors, such as limited access to health services and clean drinking water, contribute to the persistence of dengue in vulnerable communities (Bailon et al., 2025). In this context, analyzing these factors is essential to understanding the changes in the genotypic diversity of DENV-2 and improving surveillance and control strategies for the disease in the region.

Our findings may have an impact on disease control and outbreak prediction by providing insights into evolutionary dynamics in the Cajamarca region. The identification of cosmopolitan genotype fixation and the shift of the Am/As genotype after more than two decades indicate a change in viral circulation that may influence the transmissibility and clinical severity of outbreaks, as described in two independent clinical studies in Nicaragua. In these studies, the increased risk of severe DENV-2-related disease was correlated with the replacement of the NI-1 clade of Asian/American DENV-2 by a new virus clade, NI-2B (OhAinle et al., 2011; Vicente et al., 2016). By recognizing this pattern, health authorities can adjust their epidemiological surveillance strategies and design specific interventions to contain the spread of the new genotype, thus avoiding the collapse of local health systems (Vicente et al., 2016).

In turn, identifying an early diversification process in cosmopolitan strains reinforces the need for continued genomic surveillance. The accumulation of mutations, particularly the non-synonymous mutation (H158P) in the E gene, could be associated with changes in the virulence or transmission efficiency of the virus (Nonyong et al., 2023). Therefore, sequencing complete genomes will allow for a better assessment of these factors and the prediction of possible increases in the severity of outbreaks, contributing to a more efficient response since the increase in epidemic potential and disease severity are influenced by transmissibility and virulence (Ko et al., 2020). Finally, the expansion of the cosmopolitan genotype poses an emerging challenge for public health, and its detailed study will allow for the adjustment of prevention strategies, including the targeting of vector control campaigns and the optimization of health resources.

## 5 Conclusion

DENV outbreaks reported in Cajamarca are described, highlighting the emergence of new intragenotypic variants of the Am/As genotype due to local evolution and the arrival of the cosmopolitan genotype in the Cajamarca region. Both genotypes circulated together in 2020; after this period, the cosmopolitan genotype became fixed in the population and may have displaced the Am/As genotype.

Among the cosmopolitan strains detected in Cajamarca, no autochthonous variants were observed. However, the analyses conducted suggest the onset of an early diversification process, which is primarily characterized by the accumulation of synonymous mutations and a single non-synonymous mutation (H158P) in the E gene. Nonetheless, to obtain conclusive results, it is necessary to analyze complete genomes.

The expansion of the cosmopolitan genotype in Peru represents a new challenge for public health, making it necessary to implement new adjustments in prevention and control strategies. Our results provide relevant information on the changes in the diversity of DENV-2 genotypes over time in the Cajamarca region and highlight the importance of active genetic and genomic surveillance to anticipate and mitigate the future impact of this genotype in this region.

The findings provide information for planning and optimizing preventive and control measures through the implementation of epidemiological alerts, training health personnel to enhance early recognition and prevent treatment delays, and improving the surveillance system.

## 6 Limitations

The limited public information on serotype surveillance during the years when no samples could be taken, the scarce availability of public DENV-2 sequences isolated in Peru, and, above all, the absence of isolated sequences in the Cajamarca region to date have all been limiting factors for the conclusions of this study regarding the actual prevalence of the serotype and the origins of the diversity found in Cajamarca compared to other Peruvian regions, as well as for understanding the routes through which new variants were introduced at a local level.

Although it was possible to obtain a representative number of complete sequences of the DENV-2 E gene through direct sequencing from blood serum samples, limitations arose due to the viral load of the samples. As a result, a smaller number of complete sequences were obtained compared to the total number of analyzed and sequenced samples.

It is worth mentioning that despite the limitations described in this study, novel and relevant results regarding the intraserotypic diversity of DENV-2 outbreaks in Cajamarca were obtained.

## Data Availability

The authors confirm that the data are publicly available and provide access: https://doi.org/10.6084/m9.figshare.27095797.
