# Supplementary material for: Genotypic diversity and molecular characterization of DENV-2 in a Peruvian endemic region from 2016 to 2022: displacement of American/Asian genotype
Source: Front Microbiol. 2025 Apr 28;16:1558761. doi: 10.3389/fmicb.2025.1558761 (PMC12066640; doi:10.3389/fmicb.2025.1558761)
Supplement: Supplementary file 1 [file Table_1.docx]

**Table S1. Primers and probes used in this study.**

| **Real-time RT-PCR primers for DENV detection** | |
| --- | --- |
| DProbe | 5`FAM-ACAGCATATTGACGCTGGGARAGACC-3`TAMRA |
| DForward | AGGACYAGAGGTTAGAGGAGA |
| DReverse | CGYTCTGTGCCTGGAWTGAT |
| **Real-time RT-PCR primers for the detection of DENV-2** | |
| DEN-2 probe | 5`FAM-CTCTCCGAGAACAGGCCTCGACTTCAA-3`TAMRA |
| DEN-2 F | CAGGTTATGGCACTGTCACGAT |
| DEN-2 C | CCATCTGCAGCAACACCATCTC |
| **RT-PCR primers for amplification of the DENV E gene** | |
| DENV-2 PCR-F | TCGCTCCTTCAATGACAATGC |
| DENV-2 PCR-R | CAGCTCACAACGCAACCACTA |
| **DENV-2 E gene sequencing primers** | |
| D2SEQ1 | TYGCTCCTTCAATGACAATGCG |
| D2SEQ2 | ACATGCAAAAAGAACATGGAAGGA |
| D2SEQ3 | AATCCCCAYGCVAAGAAACAGGAT |
| D2SEQ4 | CCATTCGGRGACAGCTACATCAT |
| D2SEQ5 | CAGCTCACAACGCAACCACTA |
| D2SEQ6 | TGATGATGTAGCTGTCTCCGAATG |
| D2SEQ7 | CTGTGAGTGCCGTGTGCATG |
| D2SEQ8 | CAARTTTTCTGGTTGCACGACT |

**Table S2. Sequences used in the phylogenetic analysis of DENV-2.**

| **GenBank Accession Number** | **Label of Sequence** | **Isolates** | **Region of isolation** | **Country of isolation** | **Year of isolation** | **Genotype** | **Lineage** | **Variant/Sub-lineage** |
| --- | --- | --- | --- | --- | --- | --- | --- | --- |
| DQ181806 | DQ181806/Thailand/1974 | ThD2_0038_74 | - | Thailand | 1974 | Asian 1 | - | - |
| JF804027 | JF804027/Vietnam/1988 | VN/DB014/1988 | - | Vietnam | 1988 | Asian 1 | - | - |
| KX262942 | KX262942/China/2015 | DENV-2/China/YN/15DGR289(2015) | - | China | 2015 | Asian 1 | - | - |
| AF204178 | AF204178/China/1987 | strain 43/China1987 | - | China | 1987 | Asian 2 | - | - |
| JF804032 | JF804032/Guam-EEUU/2001 | GU/DB019/2001 | - | EEUU | 2001 | Asian 2 | - | - |
| MW946475 | MW946475/Tonga/1974 | TONGA-74 | - | Tonga | 1974 | American | - | - |
| DQ917242 | DQ917242/Loreto-Peru/1995 | IQT-1950 | Iquitos | Peru | 1995 | American | - | - |
| AY577439 | AY577439/Loreto-Peru/1996 | IQT2133/1996 | Iquitos | Peru | 1996 | American | - | - |
| DQ364484 | DQ364484/Jamaica/1983 | JAM_95_83 | - | Jamaica | 1983 | Asian/American | 1 | - |
| JX051767 | JX051767/Piura-Peru/2000 | OBT1012PER00 | Piura | Peru | 2000 | Asian/American | 1 | - |
| JX051770 | JX051770/Piura-Peru/2001 | OBT2093/PER/01 | Piura | Peru | 2001 | Asian/American | 1 | - |
| JX051774 | JX051774/Loreto-Peru/2002 | IQD2856/PER/02 | Loreto | Peru | 2002 | Asian/American | 1 | - |
| JX051775 | JX051775/Loreto-Peru/2002 | IQD2005/PER/02 | Iquitos | Peru | 2002 | Asian/American | 1 | - |
| JX051779 | JX051779/Madre de Dios-Peru/2007 | FMD1337/PER/07 | Madre de dios | Peru | 2007 | Asian/American | 1 | - |
| JX051777 | JX051777/Madre de Dios-Peru/2007 | FMD1346/PER/07 | Madre de dios | Peru | 2007 | Asian/American | 1 | - |
| JX051780 | JX051780/Madre de Dios-Peru/2008 | FMD2210/PER/08 | Madre de dios | Peru | 2008 | Asian/American | 1 | - |
| JX051781 | JX051781/Madre de Dios-Peru/2009 | FMD2303/PER/09 | Madre de dios | Peru | 2009 | Asian/American | 1 | - |
| JX051784 | JX051784/Madre de Dios-Peru/2009 | FMD2285PER09 | Madre de dios | Peru | 2009 | Asian/American | 2 | - |
| KC294221 | KC294221/Loreto-Peru/2010 | DENV-2/PE/IQA 2080/2010 | Iquitos | Peru | 2010 | Asian/American | 2 | - |
| KC294209 | KC294209/Loreto-Peru/2011 | DENV-2/PE/FPI01399/2011 | Iquitos | Peru | 2011 | Asian/American | 2 | - |
| JX051798 | JX051798/Tumbes-Peru/2011 | MIS1313/PER/11 | Tumbes | Peru | 2011 | Asian/American | 2 | - |
| KC294208 | KC294208/Loreto-Peru/2011 | DENV-2/PE/FPI01345/2011 | Iquitos | Peru | 2011 | Asian/American | 2 | - |
| JX051793 | JX051793/Loreto-Peru/2011 | FPI2645/PER/11 | Loreto | Peru | 2011 | Asian/American | 2 | - |
| KC847992 | KC847992/Tumbes-Peru/2012 | FPT0754/PER/12 | Tumbes | Peru | 2012 | Asian/American | 2 | - |
| KC847993 | KC847993/Tumbes-Peru/2012 | FPT0830PER12 | Tumbes | Peru | 2012 | Asian/American | 2 | - |
| MT379609 | MT379609/Loreto-Peru/2018 | FA4IQ084/PE/2018 | Iquitos | Peru | 2018 | Asian/American | 2 | - |
| MT379608 | MT379608/Loreto-Peru/2018 | FA4YU005/PE/2018 | Yurimaguas | Peru | 2018 | Asian/American | 2 | - |
| MT379611 | MT379611/Loreto-Peru/2018 | FA4IQ040/PE/2018 | Iquitos | Peru | 2018 | Asian/American | 2 | - |
| MT379586 | MT379586/Loreto-Peru/2018 | FA4YU132/PE/2018 | Yurimaguas | Peru | 2018 | Asian/American | 2 | - |
| MT379579 | MT379579/Loreto-Peru/2019 | FA4YU461/PE/2019 | Yurimaguas | Peru | 2019 | Asian/American | 2 | - |
| MT379581 | MT379581/Loreto-Peru/2019 | FA4YU226/PE/2019 | Yurimaguas | Peru | 2019 | Asian/American | 2 | - |
| PV078448 | PV078448/Cajamarca-Peru/2019 | PeCa33.11_2019 | Cajamarca | Peru | 2019 | Asian/American | 2 |  |
| ON123645 | ON123645/Piura-Peru/2021 | FPP04238/PIU-PER/2021 | Piura | Peru | 2021 | Asian/American | 2 | - |
| KY474331 | KY474331/Ecuador/2014 | 00166-P |  | Ecuador | 2014 | Asian/American | 2 | - |
| KY474317 | KY474317/Ecuador/2014 | TD-00021-S | - | Ecuador | 2014 | Asian/American | 2 | - |
| KY474311 | KY474311/Ecuador/2014 | TD-00079-P | - | Ecuador | 2014 | Asian/American | 2 | - |
| MN462633 | MN462633/Ecuador/2015 | 00240-P | - | Ecuador | 2015 | Asian/American | 2 | - |
| MN462636 | MN462636/Ecuador/2015 | 00265-P | - | Ecuador | 2015 | Asian/American | 2 | - |
| KU878564 | KU878564/Colombia/2013 | 422641_Cauca_CO_2013 | - | Colombia | 2013 | Asian/American | 2 | - |
| MK778405 | MK778405/Colombia/2015 | AC79 | - | Colombia | 2015 | Asian/American | 2 | - |
| HM488257 | HM488257/Guam-EEUU/2001 | DENV-2/GU/BID-V2950/2001 | - | Guam-EEUU | 2001 | Cosmopolitan | C | V |
| EU179857 | EU179857/Brunei/2005 | DS31-291005 | - | Brunei | 2005 | Cosmopolitan | C | V |
| MW512387 | MW512387/Singapore/2013 | SG(EHI)D2/50620Y13 | - | Singapore | 2013 | Cosmopolitan | C | V |
| KX452040 | KX452040/Malaysia/2014 | TM223 | - | Malaysia | 2014 | Cosmopolitan | C | V |
| KU517847 | KU517847/Philippines/2015 | PH-CN77-15 | - | Philippines | 2015 | Cosmopolitan | C | V |
| LC436675 | LC436675/Bangladesh/2017 | B17-1634 | - | Bangladesh | 2017 | Cosmopolitan | C | V |
| LC436672 | LC436672/Bangladesh/2017 | B17-1489/2017 | - | Bangladesh | 2017 | Cosmopolitan | C | V |
| LC436646 | LC436646/Bangladesh/2017 | 65e-S1186 | - | Bangladesh | 2017 | Cosmopolitan | C | V |
| OQ826866 | OQ826866/Bangladesh/2018 | D2-18-17 | - | Bangladesh | 2018 | Cosmopolitan | C | V |
| MN328061 | MN328061/Bangladesh/2019 | CHRF_DenV002/2019 | - | Bangladesh | 2019 | Cosmopolitan | C | V |
| OM791800 | OM791800/Madre_de_Dios/2019 | PE/DB274/2019 | Madre de dios | Peru | 2019 | Cosmopolitan | C | V |
| OM791801 | OM791801/Madre_de_Dios/2019 | PE/DB275/2019 | Madre de dios | Peru | 2019 | Cosmopolitan | C | V |
| ON123638 | ON123638/Junin-Peru/2021 | FPJ01489/JUN-PER/2021 | Junin | Peru | 2021 | Cosmopolitan | C | V |
| ON123639 | ON123639/Junin-Peru/2021 | FPJ01491/JUN-PER/2021 | Junin | Peru | 2021 | Cosmopolitan | C | V |
| OM744143 | OM744143/Brazil/2021 | GO-BC12-2021 | - | Brazil | 2021 | Cosmopolitan | C | V |
| OR039506 | OR039506/Brazil/2022 | Fiocruz-ICC_3350-22 | - | Brazil | 2022 | Cosmopolitan | C | V |
| OR138986 | OR138986/Brazil/2022 | denv2/Brazil/RS-20856/2022 | - | Brazil | 2022 | Cosmopolitan | C | V |
| ON634755 | ON634755/Brazil/2022 | NS11458 | - | Brazil | 2022 | Cosmopolitan | C | V |
| AY858036 | AY858036/Indonesia/2004 | TB16i | - | Indonesia | 2004 | Cosmopolitan | C | IV |
| KX380819 | KX380819/Singapore/2012 | D2/SG/CT24/2012 | - | Singapore | 2012 | Cosmopolitan | C | IV |
| KU517846 | KU517846/Indonesia/2014 | ID-CN18-14 | - | Indonesia | 2014 | Cosmopolitan | C | IV |
| KC762660 | KC762660/Indonesia/2007 | MKS-0084 | - | Indonesia | 2007 | Cosmopolitan | C | II |
| KM279597 | KM279597/Singapore/2012 | DC719Y12 | - | Singapore | 2012 | Cosmopolitan | C | II |
| MH985858 | MH985858/Australia/2016 | SI 2016 | - | Australia | 2016 | Cosmopolitan | C | II |
| EU179859 | EU179859/Brunei/2006 | DS09-280106 | - | Brunei | 2006 | Cosmopolitan | C | III |
| MW512376 | MW512376/Singapore/2012 | SG(EHI)D2/28912Y12 | - | Singapore | 2012 | Cosmopolitan | C | III |
| KU365902 | KU365902/Taiwan/2015 | D2/Taiwan/704TN1506b | - | Taiwan | 2015 | Cosmopolitan | C | III |
| GQ398263 | GQ398263/Indonesia/1975 | DENV-2/ID/1023DN/1975 | - | Indonesia | 1975 | Cosmopolitan | C | I |
| AB189122 | AB189122/Indonesia/1998 | 98900663 DHF DV-2 | - | Indonesia | 1998 | Cosmopolitan | C | I |
| KF744398 | KF744398/Philippines/2005 | 05-Sa-018 | - | Philippines | 2005 | Cosmopolitan | C | I |
| MW512489 | MW512489/Singapore/2018 | SG(EHI)D2/33136Y18 | - | Singapore | 2018 | Cosmopolitan | C | I |
| FJ538922 | FJ538922/India/1983 | NIV_836379 | - | India | 1983 | Cosmopolitan | B | Indian sub-continent |
| GQ252677 | GQ252677/Sri_Lanka/2003 | DENV-2/LK/BID-V2422/2004 | - | Sri_Lanka | 2003 | Cosmopolitan | B | Indian sub-continent |
| JQ955623 | JQ955623/India/2009 | RR44 | - | India | 2009 | Cosmopolitan | B | Indian sub-continent |
| KY672954 | KY672954/China/2015 | DENV-2/China/YN/JH1516(2015) | - | China | 2015 | Cosmopolitan | B | Indian sub-continent |
| MH110592 | MH110592/China/2017 | D2/CN/HZ-594/2017 | - | China | 2017 | Cosmopolitan | B | Indian sub-continent |
| GQ398258 | GQ398258/Indonesia/1975 | DENV-2/ID/1016DN/1975 | - | Indonesia | 1975 | Cosmopolitan | A | A |
| EU056810 | EU056810/Burkina_Faso/1983 | Isolate 1349/1983 | - | Burkina_Faso | 1983 | Cosmopolitan | A | A |
| KY627762 | KY627762/Burkina_Faso/2016 | 7869191/BF/2016 | - | Burkina_Faso | 2016 | Cosmopolitan | A | A |

**Table S3. DENV-2 specific antibodies and critical epitopes**

| **Targeted antibodies** | **Antibody** | **Epitope site** | **Key interacting residues** | **Neutralizing effects** | **ADE efects** | **Reference** |
| --- | --- | --- | --- | --- | --- | --- |
| DENV-2 specific epitope | 1A1D-2 | DIII lateral ridge | LR [K310] E383, P348 | Strongly neutralizes DENV-2 | NA | [DOI: 10.1128/JVI.00432-07](https://doi.org/10.1128/jvi.00432-07) |
|  | 9D12 | DIII lateral ridge | LR [K310] E383, P348 | Moderately neutralizes DENV-2 | NA | [DOI: 10.1128/JVI.00432-07](https://doi.org/10.1128/jvi.00432-07) |
|  | 3F9 | DI central | DI-centre | Neutralizes DENV-2 | NA | [DOI: 10.1371/journal.ppat.1006934](https://doi.org/10.1371/journal.ppat.1006934) |
|  | DV2-44 | Hinge DI-II | DI-II [K88, Q233, H244] | Neutralizes DENV-2 | prevent ADE | [DOI: 10.1128/JVI.01087-10](https://doi.org/10.1128/jvi.01087-10) |
|  | DV2-76 | DIII | Strand A [V309] | Neutralizes DENV-2 | prevent ADE | [DOI: 10.1128/JVI.01087-11](https://doi.org/10.1128/jvi.01087-10) |
|  | DV2-104 | DIII | Strand C-D [P334, M340, H346] | Neutralizes DENV-2 | prevent ADE | [DOI: 10.1128/JVI.01087-12](https://doi.org/10.1128/jvi.01087-10) |
|  | 3H5 | DIII | Hinge DIII-DI [298-303]; B-C loop [328-332]; [K361, V282, E383, P384 and C strand]; CD loop, FG loop [381-385] | Neutralizes DENV-2 | Marginal ADE | [DOI: 10.1038/s41590-018-0227-7 / DOI: 10.1128/JVI.79.2.1223-1231.2005](https://doi.org/10.1038/s41590-018-0227-7) |
|  | 2C8 | DIII | Hinge DIII-DI [298-303]; B-C loop [328-332]; [K361, V282, E383, P384] | Neutralizes DENV-2 | Induce ADE | [DOI: 10.4049/jimmunol.1200227](https://doi.org/10.4049/jimmunol.1200227) |
|  | DENV-290 | DII | EDII | Neutralizes DENV-2 | NA | [DOI: 10.1038/s41590-018-0227-7](https://doi.org/10.1038/s41590-018-0227-7) |
|  | DV2-48 | DI | G177 | Neutralizes DENV-2 | NA | [DOI: 10.3389/fimmu.2023.1200195](https://doi.org/10.3389/fimmu.2023.1200195) |
|  | DV2-51 | DI | Q184 | Neutralizes DENV-2 | NA | [DOI: 10.3389/fimmu.2023.1200195](https://doi.org/10.3389/fimmu.2023.1200195) |
| Cross-reactive epitope | 7F4 | EDII | DII [E67, E69 and E118] | Cross-neutralizing DENV[1-4] | Undetected | [DOI: 10.1128/JVI.01874-13](https://doi.org/10.1128/jvi.01874-13) |
|  | E53 | DII fusion loop | FL [G104, C105, G106, L107, G109, K110]; bc [C74, T76, M77, G78, E79] | Cross-neutralizing DENV[1-4] | Prevailing ADE | [DOI: 10.1128/JVI.05237-11](https://doi.org/10.1128/jvi.05237-11) |
|  | m366.6 | DIII | Strand A (K310) | Cross-neutralizing DENV[1-4] | Undetectable level | [DOI: 10.1371/journal.ppat.1007836](https://doi.org/10.1371/journal.ppat.1007836) |
|  | 3E31 | DIII | Strand A-B [314-370]; Strand E [365-370]; [Q316, H317, E368,E370] | Cross-neutralizing DENV[1-4] | No ADE | [DOI: 10.1016/j.str.2017.11.017](https://doi.org/10.1016/j.str.2017.11.017) |
|  | 4E5A | DIII | Strand D-E [358-365]; [Y360,S363] | Cross-neutralizing DENV[1-4] | Undetectable level | [DOI: 10.1016/j.cell.2015.06.057](https://doi.org/10.1016/j.cell.2015.06.057) |
|  | 4.00E+11 | DIII | Strand A [K310,E311] | Cross-neutralizing DENV[1-4] | Minor level | [DOI: 10.1371/journal.pntd.0006209](https://doi.org/10.1371/journal.pntd.0006209) |
|  | 2H12 | DIII | Strand A [314-317]; FL[K110, E114, T115, Q116,H117] | Cross-neutralizing DENV[1-4] | Minor level | [DOI: 10.4049/jimmunol.1200227](https://doi.org/10.4049/jimmunol.1200227) |
|  | 9F12 | DIII | Strand A [K305, K307,K310]; Strand B [327-331] | Cross-neutralizing DENV[1-4] | NA | [DOI: 10.1099/vir.0.006874-0](https://doi.org/10.1099/vir.0.006874-0) |
|  | N297Q-B3B9 | DII fusion loop | Fusion loop | Cross-neutralizing DENV[1-4] | Lack of ADE | [DOI: 10.7717/peerj.4021](https://doi.org/10.7717/peerj.4021) |
|  | SigN-3C | DII fusion loop and DIII | FL [G100,W101]; Strand A [K310] | Cross-neutralizing DENV[1-4] | Abrogate ADE | [DOI: 10.1038/s41541-018-0044-x](https://doi.org/10.1038/s41541-018-0044-x) |
|  | 2A10G6 | DII fusion loop | FL [98D,99R,100X,101W] | Cross-neutralizing DENV[1-4] | Undetected | [DOI: 10.1016/j.chom.2016.04.013 / DOI: 10.1371/journal.pone.0016059](https://doi.org/10.1016/j.chom.2016.04.013) |
|  | IC19 | DII-bc loop | bc [R73,G78,E79] | Cross-neutralizing DENV[1-4] | Undetected | [DOI: 10.1128/mBio.00873-13](https://doi.org/10.1128/mbio.00873-13) |
|  | d448 | DII | [D215, P219, M237,Q256, G266] | Cross-neutralizing DENV[1-4] | Undetected | [DOI: 10.1371/journal.ppat.1007716](https://doi.org/10.1371/journal.ppat.1007716) |
|  | DM25-3 | DII VLPs | FL [W101] | Cross-neutralizing DENV[1-4] | Undetected | [DOI: 10.7554/eLife.38970](https://doi.org/10.7554/elife.38970) |
|  | MZ4 | DII and DI linker | [299-306] | Neutralizes DENV-2 and ZIKV | Lesser extend | [DOI: 10.1038/s41591-019-0746-2](https://doi.org/10.1038/s41591-019-0746-2) |
| Quaternary epitope | 2D22 | Dimer of DIII and DII | Strand B [R323]; FL and bc loop | Neutralizes DENV-2 | Prevent ADE | [DOI: 10.1128/mBio.01461-15](https://doi.org/10.1128/mbio.01461-15) |
|  | 1L12 | DIII | Strand B [R323] | Neutralizes DENV-2 | NA | [DOI: 10.1371/journal.ppat.1006934](https://doi.org/10.1371/journal.ppat.1006934) |
|  | A11, B7, C8, C10 | Dimer of DI and DII | DI [148-159, N153 glycan], FL loop [97-106], ij loop [246-249] | Neutralizes DENV-2 | NA | [DOI: 10.1038/nature14130](https://doi.org/10.1038/nature14130) |
|  | J8, J9 | DI | DI [K47,H149,V151,F279] | Cross-neutralizing DENV[1-4] | No ADE | [DOI: 10.7554/eLife.52384](https://doi.org/10.7554/eLife.52384) |
| NA: Not available | |  |  |  |  |  |

**Table S4. Am/As Lineage 2 average sequence identity.**

| Sequence group | Year | DQ364484 Jamaica_1983 | | JX051784  Madre de Dios_2009 | | JX051798 Tumbes_2011 | | |
| --- | --- | --- | --- | --- | --- | --- | --- | --- |
|  |  | nt (%Id) | aa (%Id) | nt (%Id) | aa (%Id) | nt (%Id) | aa (%Id) |  |
| Cajamarca Am/As II “b” | 2016 | 97.50 | 98.70 | 97.50 | 99.30 | 99.50 | 99.70 |  |
| Cajamarca Am/As II “b” | 2017 | 97.50 | 98.45 | 97.38 | 99.05 | 99.38 | 99.45 |  |
| Cajamarca Am/As II “a” | 2020 | 96.10 | 98.10 | 98.40 | 99.50 | 96.55 | 99.10 |  |
| *Piura Am/As II “a” | 2021 | 96.00 | 97.90 | 98.30 | 99.30 | 96.50 | 98.90 |  |
| *Loreto Am/As II “a” | 2018 | 96.20 | 98.05 | 98.65 | 99.45 | 96.80 | 99.05 |  |
| *Loreto Am/As II “a” | 2019 | 96.40 | 98.00 | 98.50 | 99.40 | 96.70 | 99.00 |  |

* Sequence group for non-isolated Am/As lineage 2 genotype in this study.

Cajamarca: Set of isolated sequences in this region which are grouped by variants as indicated.

DQ364484 Jamaica_1983: Prototype sequence of Am/As genotype.

JX051784 Madre de Dios_2009: Ancestral Am/As lineage 2 strain from southeastern side.

JX051798 Tumbes_2011: Ancestral Am/As lineage 2 strain from northern side.

**Table S5.** **Cosmopolitan average sequence identity.**

| Sequence group | Year | GQ398263 Indonesian_1975 | | LC436672 Bangladesh_2017 | | OM791801 Madre de Dios_2019 | |
| --- | --- | --- | --- | --- | --- | --- | --- |
|  |  | nt (%Id) | aa (%Id) | nt (%Id) | aa (%Id) | nt (%Id) | aa (%Id) |
| *Madre de Dios | 2019 | 96.55 | 99.30 | 99.55 | 100.00 | - | - |
| Cajamarca | 2020 | 96.30 | 99.30 | 99.30 | 100.00 | 99.70 | 100.00 |
| Cajamarca | 2021 | 96.37 | 99.21 | 99.42 | 99.93 | 99.71 | 99.93 |
| Cajamarca (H158P) | 2021 | 96.20 | 99.06 | 99.28 | 99.66 | 99.60 | 99.66 |
| Cajamarca | 2022 | 96.30 | 99.30 | 99.32 | 100.00 | 99.66 | 100.00 |
| **Junin | 2021 | 96.55 | 99.30 | 99.45 | 100.00 | 99.80 | 100.00 |

* Ancestral sequences of cosmopolitan genotype that were introduced in Peru.

** Group of sequences of cosmopolitan genotype that were not isolated in this study.

Cajamarca: Set of sequences that were isolated in this region and are grouped as indicated.

GQ398263 Indonesian_1975: Prototype sequence of cosmopolitan genotype.

LC436672 Bangladesh_2017: Most likely ancestral sequence of Peruvian cosmopolitan strain.

OM791801 Madre de Dios_2019: First cosmopolitan sequence that was isolated in Peru.
