# Supplementary figures and images for: Genotypic diversity and molecular characterization of DENV-2 in a Peruvian endemic region from 2016 to 2022: displacement of American/Asian genotype
Source: Front Microbiol. 2025 Apr 28;16:1558761. doi: 10.3389/fmicb.2025.1558761 (PMC12066640; doi:10.3389/fmicb.2025.1558761)

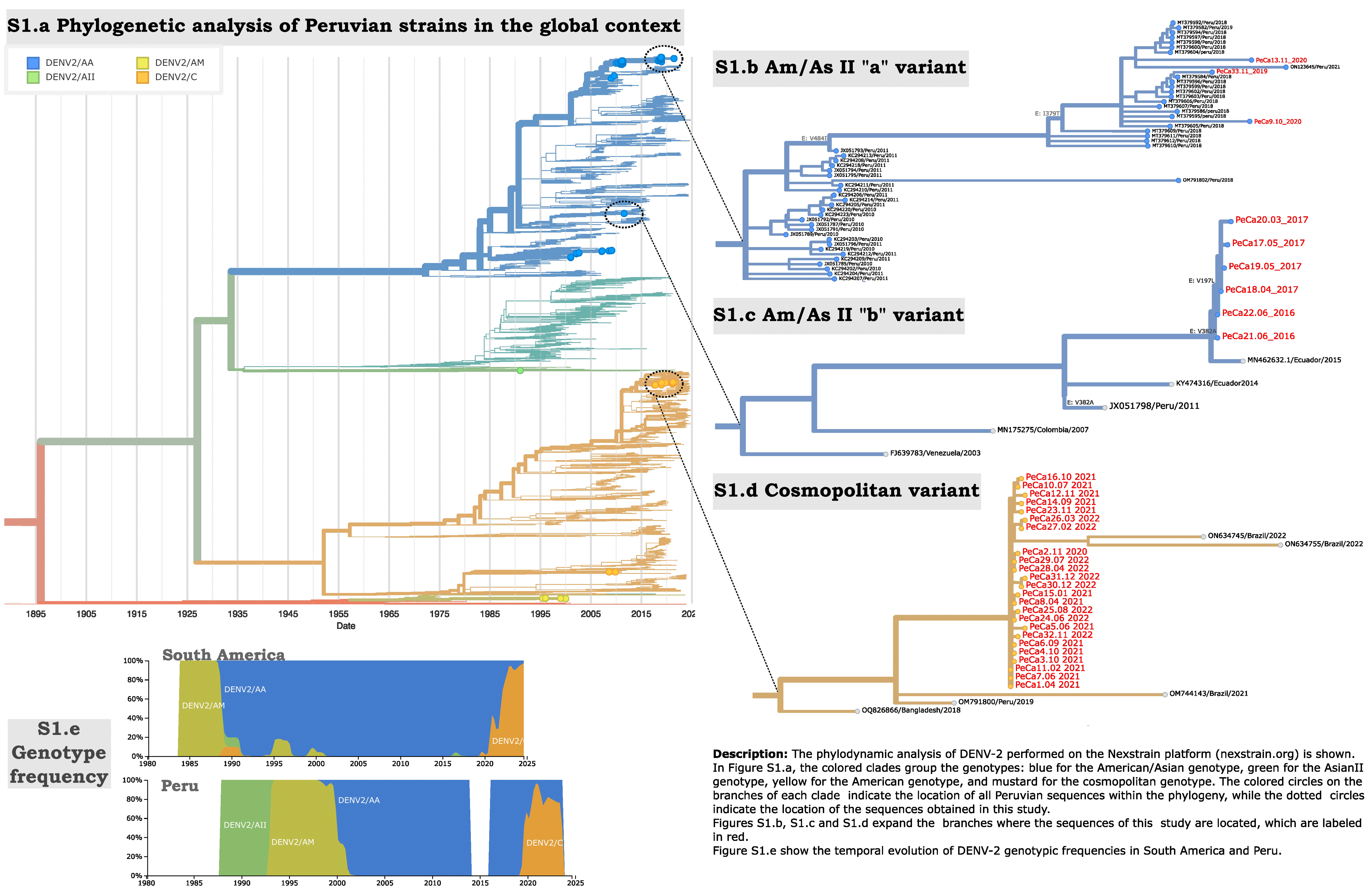

Supplement: Supplementary file 2 [file Image_1.jpeg]
